# Efficient CRISPR-Cas9-Mediated Knock-In of Composite Tags in Zebrafish Using Long ssDNA as a Donor

**DOI:** 10.3389/fcell.2020.598634

**Published:** 2021-02-11

**Authors:** Deshani C. Ranawakage, Keita Okada, Kota Sugio, Yuya Kawaguchi, Yuki Kuninobu-Bonkohara, Takuya Takada, Yusuke Kamachi

**Affiliations:** School of Environmental Science and Engineering, Kochi University of Technology, Kochi, Japan

**Keywords:** CRISPR-Cas9, long ssDNA donor, knock-in, endogenous tagging, SoxB1, composite tag

## Abstract

Despite the unprecedented gene editing capability of CRISPR-Cas9-mediated targeted knock-in, the efficiency and precision of this technology still require further optimization, particularly for multicellular model organisms, such as the zebrafish (*Danio rerio*). Our study demonstrated that an ∼200 base-pair sequence encoding a composite tag can be efficiently “knocked-in” into the zebrafish genome using a combination of the CRISPR-Cas9 ribonucleoprotein complex and a long single-stranded DNA (lssDNA) as a donor template. Here, we targeted the *sox3*, *sox11a*, and *pax6a* genes to evaluate the knock-in efficiency of lssDNA donors with different structures in somatic cells of injected embryos and for their germline transmission. The structures and sequence characteristics of the lssDNA donor templates were found to be crucial to achieve a high rate of precise and heritable knock-ins. The following were our key findings: (1) lssDNA donor strand selection is important; however, strand preference and its dependency appear to vary among the target loci or their sequences. (2) The length of the 3′ homology arm of the lssDNA donor affects knock-in efficiency in a site-specific manner; particularly, a shorter 50-nt arm length leads to a higher knock-in efficiency than a longer 300-nt arm for the *sox3* and *pax6a* knock-ins. (3) Some DNA sequence characteristics of the knock-in donors and the distance between the CRISPR-Cas9 cleavage site and the tag insertion site appear to adversely affect the repair process, resulting in imprecise editing. By implementing the proposed method, we successfully obtained precisely edited *sox3*, *sox11a*, and *pax6a* knock-in alleles that contained a composite tag composed of FLAGx3 (or PAx3), Bio tag, and HiBiT tag (or His tag) with moderate to high germline transmission rates as high as 21%. Furthermore, the knock-in allele-specific quantitative polymerase chain reaction (qPCR) for both the 5′ and 3′ junctions indicated that knock-in allele frequencies were higher at the 3′ side of the lssDNAs, suggesting that the lssDNA-templated knock-in was mediated by unidirectional single-strand template repair (SSTR) in zebrafish embryos.

## Introduction

The ability to achieve precise and sequence-specific genome editing using the prokaryotic CRISPR-Cas9 system has revolutionized genomic engineering, enabling an unprecedented potential to modify the genomes of almost any organism (Mali et al., 2013; Ran et al., 2013; Barman et al., 2020). In the CRISPR-Cas9 system, the programmable guide RNA (gRNA) complex consisting of CRISPR RNA (crRNA) and a trans-activating CRISPR RNA (tracrRNA) or chimeric single gRNA (sgRNA) directs the Cas9 endonuclease to the genomic target site that is complementary to the crRNA and is associated with a protospacer adjacent motif (PAM) (Mali et al., 2013). Upon recognition of the target DNA sequence, Cas9 mediates the cleavage of target DNA upstream of PAM to create a double-strand break (DSB). This DSB is then repaired by cellular machinery through either the non-homologous end joining (NHEJ) or the homology-directed repair (HDR) pathways (Jasin and Haber, 2016; Salsman et al., 2017; Gallagher and Haber, 2018; Barman et al., 2020). NHEJ is the predominant repair pathway in higher eukaryotic cells; however, this repair mechanism is considered imprecise given that it simply re-joins the two broken ends, which often leads to disruptive insertions and deletions (indels) at the target loci. Therefore, the CRISPR-Cas9 system allows for the efficient creation of gene knock-outs by producing indels in a coding exon. In contrast, the HDR pathway is a more precise mechanism, although far less efficient than NHEJ. Notably, HDR requires a DNA template, in the form of either a sister chromatid, which is naturally available during the S and G2 phases of the cell cycle, or an exogenous artificial DNA (Salsman et al., 2017). Genome editing technology enables the precise insertion of an exogenous sequence at the cleavage site (i.e., a so-called knock-in) by supplying an exogenous repair template that contains homology arms flanking the cleavage site (Salsman et al., 2017; Lee et al., 2018). The repair template sequences can be designed for various types of genome modifications including single nucleotide substitutions, as well as epitope tag knock-ins (e.g., FLAG, HA) and larger fluorescent protein knock-ins (Peng et al., 2014; Li et al., 2016; Bukhari and Müller, 2019). Nonetheless, HDR-mediated knock-in experiments can result in undesirable indels, which co-occur with the targeted insertions. This ultimately results in a low success rate when attempting to generate knock-in alleles in multicellular model organisms, such as the zebrafish (*Danio rerio*) (Boel et al., 2018; Cornet et al., 2018; Prykhozhij and Berman, 2018; Liu et al., 2019).

The design of the donor template has been reported to be crucial to achieve efficient HDR-mediated knock-ins (Bollen et al., 2018; Ryu and Kim, 2019). In cultured cell studies, the use of long single-stranded DNA (lssDNA) as the donor DNA template has recently been shown to be more effective in HDR-based genome editing due to its lower cytotoxicity and higher integration specificity in addition to similar or even higher genome insertion efficiency than that of double-stranded DNA (dsDNA) templates, such as plasmids and polymerase chain reaction (PCR) fragments (Roth et al., 2018; Li et al., 2019). lssDNA donors have been reported to exhibit superior specificity for on-target integration, whereas dsDNA donors tend to render high levels of off-target integration likely through non-homologous integration at unwanted sites of DSBs (Roth et al., 2018; Li et al., 2019). Furthermore, mouse and rat genome editing studies have demonstrated the efficiency of lssDNAs as knock-in donors, both for insertion and for gene replacement (Yoshimi et al., 2016; Quadros et al., 2017; Miura et al., 2018; Miyasaka et al., 2018). However, the chemical synthesis of lssDNAs can be prohibitively costly, whereas shorter single-strand oligodeoxynucleotides (ssODNs, <200 bases) can be chemically synthesized at a moderate cost. Moreover, lssDNAs synthesis *via* Easi-CRISPR and plasmid nicking followed by denaturation is generally considered labor- and time-intensive (Yoshimi et al., 2016; Miura et al., 2018), and therefore the use of lssDNAs for knock-in experiments is relatively uncommon despite the potential advantages of this technique. Except for a recent study by Bai et al. (2020), the use of lssDNAs to perform knock-in studies in zebrafish had not been previously reported in the literature.

ssDNAs exhibit two different strand orientations (“target” or “non-target”) depending on gRNA placement. The target strand corresponds to the strand that is bound by the gRNAs, whereas the non-target strand is unbound and contains the PAM sequence. Previous knock-in studies that used short ssDNAs as donor templates suggest that strand selection critically affects knock-in efficiency. Furthermore, these studies reported that the lengths of homology arms are also critical determinants of knock-in efficiency when using ssDNA donors (Hwang et al., 2013; Richardson et al., 2016). However, neither of these experimental variables for the use of lssDNA donors has been properly examined.

Different transcription factor combinations determine cell-type identity by establishing specific gene regulatory networks. Sox and Pax family transcription factors are examples of such regulators and are often involved in determining the fates of various progenitor and stem cells (Kamachi and Kondoh, 2013; Blake and Ziman, 2014). For instance, the SoxB1 group members are known to play important roles in the regulatory networks of embryonic stem cells and neural progenitor cells (Sarkar and Hochedlinger, 2013). The SoxB1 group comprises Sox1a/1b/2/3/19a/19b in zebrafish and Sox1/2/3 in amniotes, all of which share amino acid sequence similarities along their entire lengths (Okuda et al., 2006), which results in potential cross-reactivity problems when using their antibodies for functional analyses.

Epitope tagging with short peptides and subsequent use of epitope-specific antibodies is a promising strategy to circumvent the aforementioned cross-reactivity problems or even the unavailability of specific antibodies, which is often the case in zebrafish research (Brizzard, 2008; Partridge et al., 2016). Two or more different tags can be combined to generate a composite tag to effectively increase their functionality for protein detection and purification (Li, 2010). For instance, epitope and affinity tags, such as the His and Bio tags (i.e., polyhistidine and biotin acceptor domain tags, respectively), are often combined for tandem affinity purification. Furthermore, the newly developed HiBiT tag enables highly sensitive protein detection through NanoLuc luciferase complementation and can be coupled with other tags (Madsen and Semple, 2019; Ranawakage et al., 2019).

Our study sought to develop an efficient method to precisely knock-in a composite tag sequence at the 3′ or 5′ end of the coding sequence of a zebrafish gene of interest using the CRISPR-Cas9 genome editing tool. Using the *sox3*, *sox11a*, and *pax6a* genes as knock-in targets, we demonstrated that a few hundred base-pair sequences encoding a composite tag can be efficiently and precisely knocked-in into the zebrafish genome using the CRISPR-Cas9 ribonucleoprotein (RNP) complex with a lssDNA donor template. Specifically, we demonstrated that the proper choice of lssDNA strands, length of the 3′ homology arm, and distance from the DSB to the knock-in site are critical for efficient and precise knock-in. With this method, we successfully obtained knocked-in *sox3*, *sox11a*, and *pax6a* alleles that contained a composite tag composed of FLAGx3 (or PAx3), Bio tag, and HiBiT tag (or His tag) with moderate to high germline transmission rates.

## Results

### Design of Composite Tags for Knock-In Experiments

**FIGURE 1:**
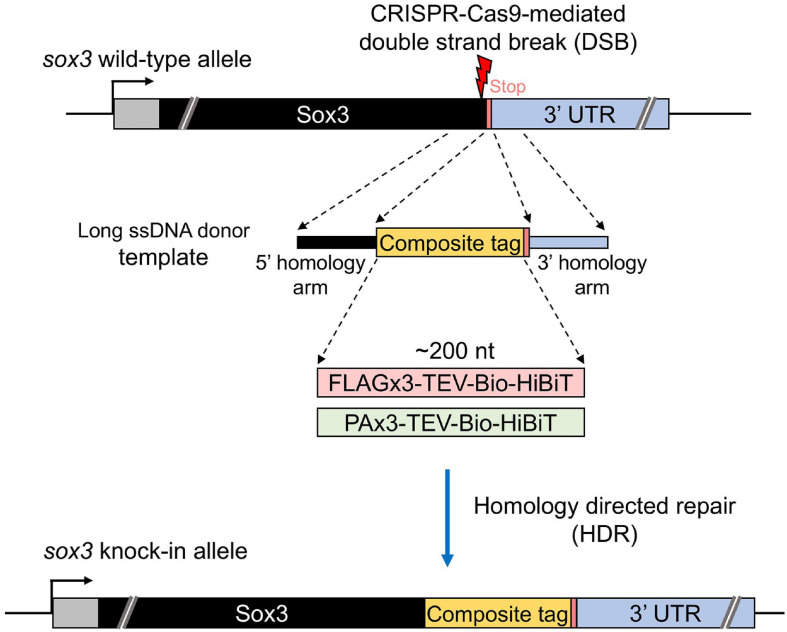
Design of composite tag knock-in through the CRISPR-Cas9 system. In this design, an ∼200-nt length composite that contains either a FLAG or PA epitope tag in its trimeric form followed by a tobacco etch virus (TEV) protease cleavage site, a biotin acceptor domain (Bio tag), and a C-terminus HiBiT peptide tag was knocked into the 3′ end of the coding sequence of the *sox3* gene. A long ssDNA donor fragment that contains the composite tag flanked at both ends by the homology arms that corresponds to the CDS upstream from the stop codon (5′ homology arm) and the 3′ UTR downstream from the stop codon (3′ homology arm) of the *sox3* gene was used as a template to induce the homology-directed repair (HDR) mechanism after the CRISPR-Cas9-mediated double-strand break (DSB).

Our study aimed to develop an efficient method to precisely knock-in an approximately 200-nt-long composite tag sequence at the 3′ or 5′ end of the coding sequence of a zebrafish gene of interest using the CRISPR-Cas9 genome editing tool (Figure 1). This composite tag encodes either the FLAG or PA epitope tags in their trimeric form, as well as the biotin acceptor domain (Bio tag), and the HiBiT peptide tag (Ranawakage et al., 2019). For this purpose, we first selected the SoxB1 transcription factor gene *sox3* as a knock-in target, as we had previously demonstrated through mRNA injection that the Sox3 protein that was C-terminally tagged with the FLAG composite tag was stably expressed in zebrafish embryos (Ranawakage et al., 2019).

**FIGURE 2:**
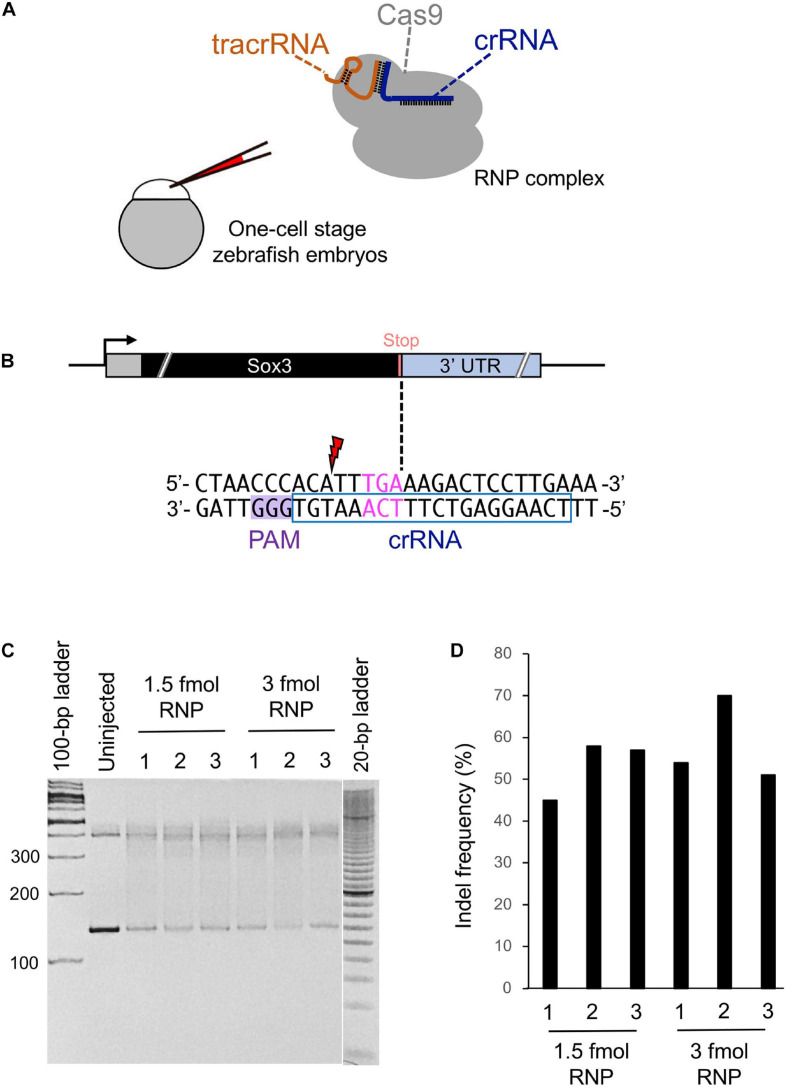
Cleavage efficiency of the selected crRNA. **(A)** Schematic illustration of the CRISPR-Cas9 ribonucleoprotein (RNP) complex and injection into a one-cell stage zebrafish embryo. **(B)** Candidate crRNA location and sequence. Double-strand break occurs 2 bases upstream from the stop codon for the selected crRNA. **(C)** Heteroduplex mobility assay (HMA) to evaluate the cleavage efficiency of crRNA. A 1.5 or 3 fmol RNP complex was microinjected, and genomic DNA was extracted from three pools of five embryos each at 1 dpf. Primers used for HMA are listed in Supplementary Table 1. **(D)** Percentage of indel mutations by Inference of CRISPR Edits (ICE) analysis.

A CRISPR-Cas9 complex that cuts DNA in the immediate vicinity of the intended editing site with high on-target cleavage activity is very likely a prerequisite for efficient knock-in (Paquet et al., 2016). Therefore, we chose a CRISPR-Cas9 system that utilizes an *in vitro* assembled RNP complex that consists of three components: two synthetic RNA oligonucleotides (i.e., a target-specific crRNA and a universal tracrRNA, both of which are chemically modified and length-optimized variants of native gRNAs), and a high-fidelity recombinant *Streptococcus pyogenes* Cas9 protein that was developed by Integrated DNA Technologies, Inc. (IDT) (Figure 2A). Although CRISPR-Cas9 RNP complexes can be assembled using sgRNAs synthesized *in vitro* with phage RNA polymerases (Jinek et al., 2012), this approach requires the presence of guanine nucleotides at the 5′ end of sgRNAs, which limits target site selection. In contrast, the use of an RNA system composed of chemically synthesized crRNA and tracrRNA allows for the selection of any PAM sequence (NGG) close to the knock-in site because it eliminates the need for guanine nucleotides at the 5′ end of crRNA. A recent study has revealed that the aforementioned crRNA/tracrRNA system is more active than the sgRNA systems because the supernumerary 5′ guanines in sgRNA interfere with cleavage activity, thereby diminishing the mutagenesis activity of RNP (Hoshijima et al., 2019). Additionally, we specifically injected Cas9 protein instead of Cas9 mRNA into one-cell stage zebrafish embryos expecting that it would immediately act upon the injected embryos during their early cleavage stages, which would enhance knock-in efficiency.

While searching for the PAM site closest to the stop codon of the *sox3* gene, we found a candidate crRNA that causes the CRISPR-Cas9 RNP complex to cleave the *sox3* gene 2 bp upstream of the intended tag insertion site (Figure 2B). Therefore, a heteroduplex mobility assay (HMA) was performed to examine the target site cleavage efficiency of CRISPR-Cas9 with this selected crRNA. At first, the RNP complex (1.5 or 3 fmol) was microinjected into the cytoplasm of one-cell stage embryos, after which genomic DNA was extracted at 24 h post-fertilization (hpf). Afterward, the target region was amplified via PCR, and the generated homoduplexes and heteroduplexes were separated via polyacrylamide gel electrophoresis (PAGE) (Ota et al., 2013). On the PAGE gel, DNA smears indicative of indel-derived heteroduplexes were observed only for the CRISPR-Cas9-injected embryos, indicating the efficient cleavage of the genome when using this crRNA (Figure 2C). Moreover, the percentage of modified genomes with indels was assessed by analyzing the Sanger sequence traces of the target region PCR amplicons using the Inference of CRISPR Edits (ICE) sequencing analysis tool^1^ (Hsiau et al., 2018). Through this method, similar indel rates were observed for 1.5 and 3 fmol RNP complex injections with averages of 53 and 58%, respectively (Figure 2D). Therefore, a 1.5 fmol amount of RNP complex was used in subsequent genome editing procedures to avoid unwanted off-target effects caused by excessive RNP use.

### Effect of lssDNA Strand Choice on Knock-In Efficiency

**FIGURE 3:**
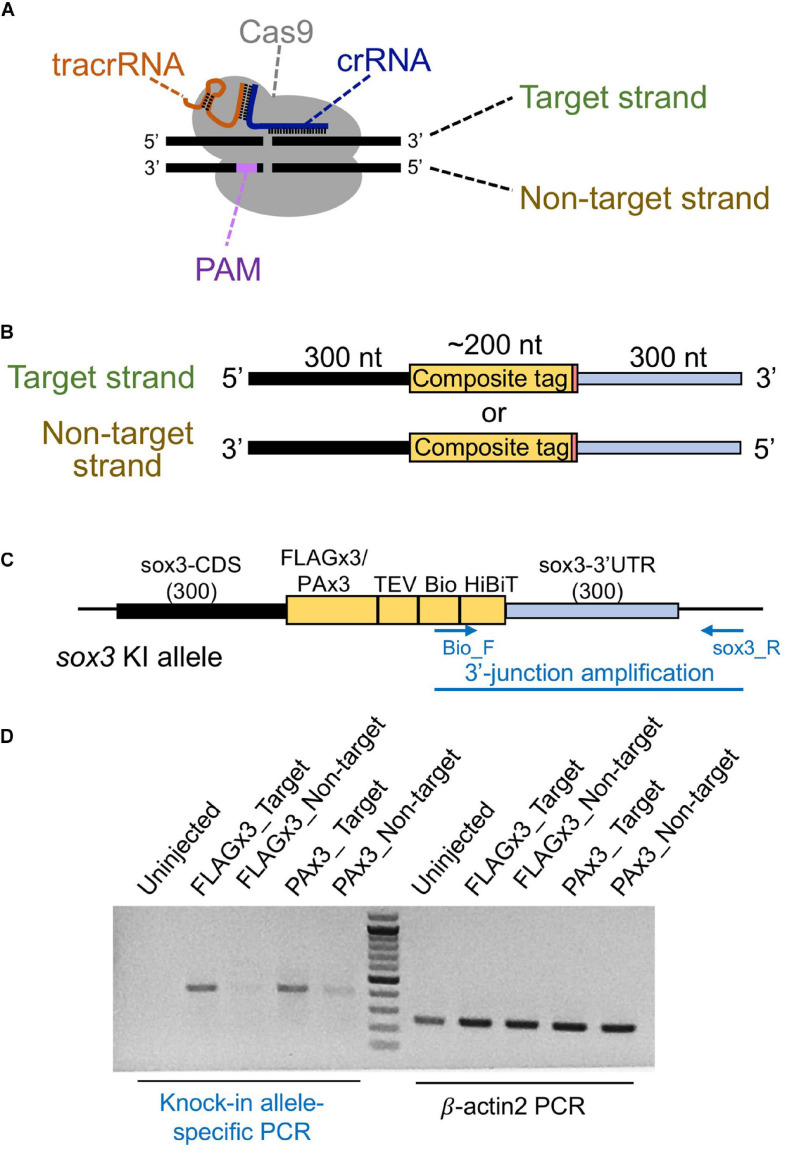
Effect of the lssDNA strand choice on knock-in efficiency. **(A)** Definition of the target and non-target strands in relation to the CRISPR-Cas9 complex. The strand that is complementary to the crRNA sequence is referred to as the target strand. **(B)** The target and non-target strands of lssDNA used as a donor template. Each lssDNA was microinjected with 1.5 fmol of the RNP complex into the cytoplasm of one-cell stage zebrafish embryos, and genomic DNA was extracted from 20-embryo pools. **(C)** Schematic illustration of the *sox3* knock-in allele and knock-in allele-specific PCR. **(D)** Agarose gel image showing the PCR amplicons of knock-in allele-specific PCR at the 3′ junction of the integration and the β*-actin2* gene-specific PCR (control) to confirm DNA integrity.

Different types of donor DNA templates can be used for homology-directed DSB repair, including plasmid DNA, dsDNA/PCR fragments, or ssDNA; however, knock-in efficiency can be significantly affected by these donor types (Ryu and Kim, 2019). Given that the use of lssDNA donor templates has been shown to increase CRISPR-mediated knock-in efficiency compared with dsDNA templates in mouse and rat experiments (Yoshimi et al., 2016; Quadros et al., 2017; Miura et al., 2018; Miyasaka et al., 2018), we first examined its applicability to zebrafish knock-in experiments. The lengths of the DNA fragments of the composite tags encoding FLAGx3-Bio-HiBiT and PAx3-Bio-HiBiT are 198 and 237 bp, respectively, and the total donor template length, with the addition of the 5′ and 3′ homology arms, exceeds the size limit of standard chemical DNA synthesis. Therefore, lssDNAs were prepared from plasmids using nicking enzymes (Yoshimi et al., 2016; Miyasaka et al., 2018). In our initial donor template design, the composite tag sequence was flanked on one side by the 300-nt homology arms of the *sox3* coding sequence (CDS) and on the other by the *sox3* 3′ UTR region aiming to insert the composite tags at the 3′ end of the *sox3* CDS (Figures 3B,C). The ssDNA donor templates can be either the target strand (complementary to and bound by the crRNA) or the PAM-containing non-target strand (Figure 3B). Importantly, previous studies using short ssDNA as a donor template have suggested that strand choice affects knock-in efficiency (Hwang et al., 2013). Thus, we first examined the efficiency of both strands by injecting either the target or non-target lssDNA strands into the cytoplasm of one-cell stage embryos along with the CRISPR-Cas9 RNP complex. Genomic DNA from 1 day post-fertilization (dpf) embryos was isolated from 20-embryo pools, and knock-in allele-specific PCR was performed at the 3′ junction of the insertion. The knock-in efficiency of the target strand was found to be superior for both FLAGx3 and PAx3 composite tag insertions (Figure 3D), indicating that the lssDNA strand choice was critical to ensure a high knock-in efficiency rate.

### Donor DNA Template Type Comparisons

**FIGURE 4:**
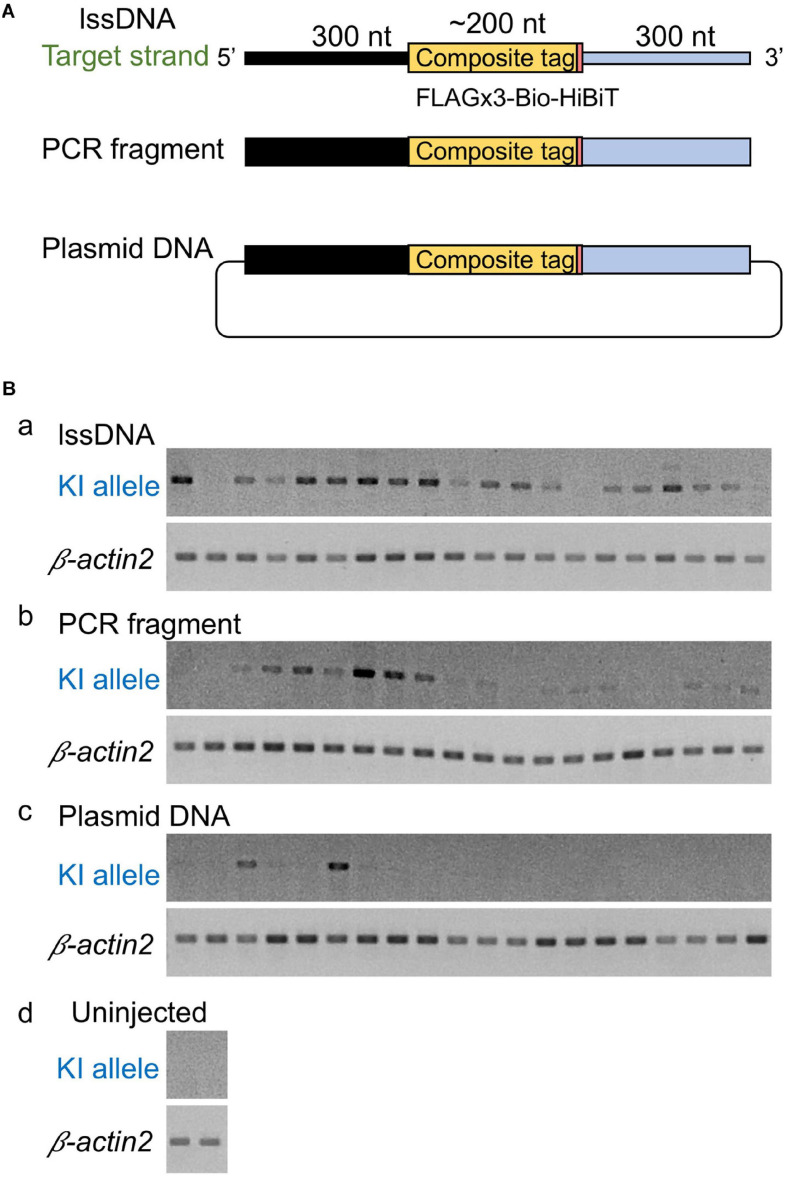
Comparison of donor DNA template types. **(A)** Donor DNA templates used in this comparison. **(B)** Knock-in (KI) allele-specific PCR amplification for the 5′ junction. lssDNA **(a)**, PCR fragments **(b)**, or plasmid DNA **(c)** was microinjected with 1.5 fmol of the RNP complex into the cytoplasm of one-cell stage zebrafish embryos, and genomic DNA was extracted from 20 individual zebrafish embryos. As a negative control, knock-in allele-specific PCR amplification was performed using uninjected zebrafish embryos **(d)**. β*-actin2* gene-specific PCR was performed to confirm DNA integrity.

Although the lssDNA donor templates were found to be effective in CRISPR-Cas9-mediated knock-ins as described above, it has also been reported that dsDNA donors are more efficient than ssDNA for the introduction of triple base substitutions into the zebrafish genome (Zhang et al., 2018). Furthermore, PCR-amplified dsDNA was also successfully applied as a knock-in donor in mouse embryos (Yao et al., 2018). Therefore, our study also sought to examine the efficiency of dsDNA as a donor. To achieve this, similar to the lssDNA donor template, we produced a PCR fragment including the FLAGx3-Bio-HiBiT composite tag sequence flanked by the 300-nt homology arms on both ends. Afterward, we compared the efficiency of this PCR fragment to the efficiency of the lssDNA template, as well as that of plasmid DNA, *via* knock-in allele-specific PCR using DNA from individual injected embryos. Interestingly, the lssDNA donor template exhibited the highest knock-in efficiency rate (∼90%; 18/20 embryos) (Figure 4). Although using the PCR fragment as a donor template showed a relatively high knock-in efficiency of ∼75% (15/20 embryos), the resulting band intensities were lower than those for the lssDNA donor in most samples. In contrast, the plasmid DNA donor template exhibited the lowest knock-in efficiency (∼15%; 3/20 embryos). It is worth noting that the lssDNA template exhibited little toxicity to the embryos and resulted in a considerable higher survival rate at 48 hpf compared with the PCR fragment and plasmid DNA donor templates, even though we injected the same mass (not molar) amount of DNA (75 pg) regardless of the template types, which is consistent with previous cell-based studies (Roth et al., 2018). This suggests that one benefit of the use of lssDNA may be that template copy numbers can be increased without toxic effects. Taken together, these results suggest that lssDNA may provide substantial advantages when used as a donor template for composite tag sequence knock-in experiments.

### Effect of lssDNA Donor Template Homology Arm Length on Knock-In Efficiency

**FIGURE 5:**
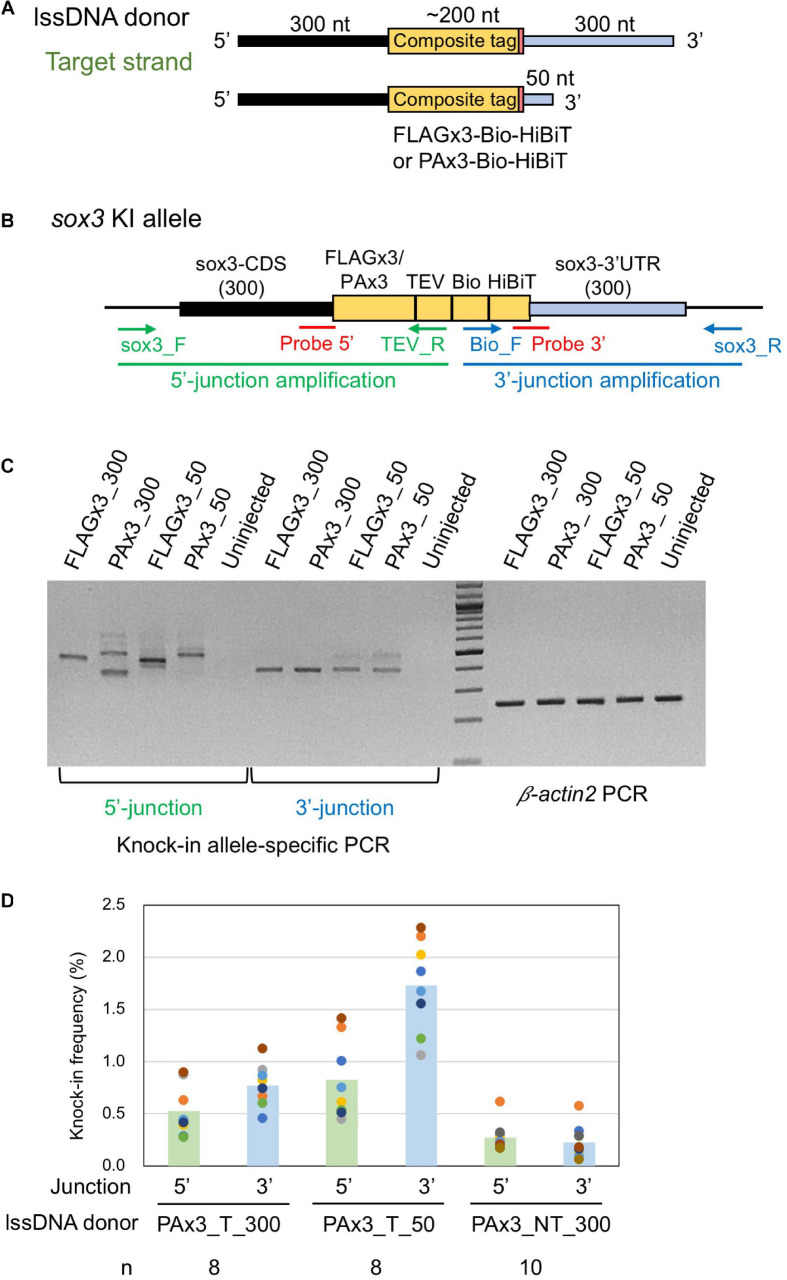
Effect of lssDNA 3′ homology arm length on knock-in efficiency. **(A)** Donor lssDNA templates with different 3′ homology arm lengths used for comparison. **(B)** Schematic illustration of the *sox3* knock-in allele and knock-in allele-specific PCRs for 5′ and 3′ junctions. Each lssDNA was microinjected with 1.5 fmol of the RNP complex into the cytoplasm of one-cell stage zebrafish embryos, and genomic DNA was extracted from 20-embryo pools. **(C)** Agarose gel image showing the PCR amplicons of knock-in allele-specific PCRs and the β*-actin2* gene-specific PCR (control) to confirm DNA integrity. **(D)** Knock-in allele-specific qPCRs for 5′ and 3′ junctions using the hydrolysis probes shown in **(B)**. The vertical bars represent the means of 8–10 replicates, each of which consists of a pooled sample of 10 injected embryos and is shown as a colored circle.

The structure of the ssDNA donor template has been shown to play an important role in determining knock-in efficiency. For instance, Richardson et al. (2016) showed that a target ssDNA donor strand with 91-nt 5′ and 36-nt 3′ homology arms resulted in the highest HDR efficiency when introducing a three-nucleotide mutation into the genome of cultured human cells (Richardson et al., 2016). Another study revealed that a 97-nt ssDNA donor template with a 30-nt 3′ homology arm exhibited efficient HDR for single-nucleotide substitution regardless of strand orientation (Liang et al., 2017). These results have been further explored through a modeling approach, which suggested that a shorter 3′ homology arm may facilitate the binding of the 3′ region of the donor template to the protruding single-stranded region, which results from resection by 5′–3′ exonucleases after DSB (Liang et al., 2017). In contrast, when using lssDNA donor templates for GFP gene integration, longer homology arms (300–700 nt) rendered the highest knock-in efficiency (Li et al., 2019), suggesting that optimal donor length might be dependent on the target locus, homology arm sequences and/or intended knock-in sequences. Given the lack of established guidelines to select an optimal donor length, we also created lssDNA target strand structures with a short 50-nt 3′ homology arm and examined its effect on knock-in efficiency (Figure 5). The 5′ homology arm length was kept at 300 nt because the use of an extended 5′ homology arm has been suggested to prevent the degradation of the 5′ end of lssDNA *via* endogenous exonuclease activity, which may result in incomplete HDR (Yoshimi et al., 2016).

lssDNA donor templates containing FLAGx3-Bio-HiBiT or PAx3-Bio-HiBiT with two different 3′ homology arm lengths were injected along with the CRISPR-Cas9 RNP complex into one-cell stage zebrafish embryos. Afterward, knock-in allele-specific PCRs were performed for both insertion junctions to compare knock-in efficiency. As shown in Figure 5C, the lssDNA donors with the 50-nt 3′ homology arm rendered comparable PCR bands to those with the 300-nt 3′ homology arm, suggesting that the shorter 3′ homology arm performs at least as well as the longer one.

Hydrolysis probe-based quantitative PCR (qPCR) assays for specific alleles were conducted to quantitatively assess knock-in efficiency. To achieve this, we designed the probes at the 5′ and 3′ junction sites to preferentially detect precisely edited knock-in alleles (Figure 5B). For these assays, the three lssDNA donor structures for the PAx3-Bio-HiBiT knock-in were each injected again into approximately 100 embryos, after which genomic DNA was extracted from 10-embryo pools at approximately 24–26 hpf. We first determined the copy numbers of the knock-in alleles by qPCR, after which we normalized them to the entire genomic DNA to obtain knock-in allele frequencies (reported as the percentage of the total). The results of the qPCR-based assays indicated that the target strand donor with the 50-nt 3′ homology arm (PAx3_T_50) exhibited higher knock-in efficiency than that with the 300-nt 3′ homology arm (PAx3_T_300). Moreover, the non-target strand donor (PAx3_NT_300) resulted in the lowest frequency of knock-in alleles, which was consistent with the above-described results (Figure 3). Interestingly, the knock-in allele frequencies were higher at the 3′ side of the lssDNA donors (i.e., the 3′ junction for the target strand donors and 5′ junction for the non-target strand donor). This asymmetric knock-in efficiency may be explained by the unidirectional nature of DSB repair mediated by single-strand template repair (SSTR) (see the section “Discussion”).

### Screening for Germline Transmission of lssDNA-Mediated Knock-Ins

Encouraged by the knock-in efficiency of lssDNA donor templates in the somatic cells of injected zebrafish embryos, we then examined their knock-in efficiency in germline cells using the four above-mentioned lssDNA donor templates (Figure 5A). More than 100 embryos injected with one of the four templates along with the CRISPR-Cas9 RNP complex were raised to adulthood and tested for germline transmission of the composite tag sequence integration. To facilitate germline transmission screening, the randomly selected F0 fish were first in-crossed pairwise, after which pooled F1 embryos were analyzed to evaluate germline composite tag integration efficiency. Both the 5′- and 3′ junctions of the insertion were examined by knock-in allele-specific PCR (Figure 5B and Supplementary Figure 1). For the PCR positive samples, the PCR products were sequenced to characterize the integration events.

To examine the germline transmission rate of the FLAGx3 composite tag knock-in, 37 F0 fish that had been injected with the 300-nt 3′ homology arm lssDNA donor template were screened. Seven in-crossed pairs were found to be positive for knock-in allele-specific PCRs, indicating a 19% minimum germline transmission rate (Table 1). As confirmed by PCR product sequencing, out of these seven pairs, one fish pair contained the correct integration at both the 3′ and 5′ junctions. By out-crossing this pair with wild-type fish, one male fish (#9) was identified as the correct knock-in founder. The six remaining fish pairs exhibited incorrect integration, either at the 5′ or 3′ junctions or both, due to sequence duplications and indels (Supplementary Figure 1). Afterward, the germline transmission rate of FLAGx3 composite tag knock-ins was examined using the lssDNA donor template with the 50-nt 3′ homology arm. A total of 28 F0 fish were then screened, of which eight in-crossed pairs were positive for knock-in allele-specific PCRs; this corresponded to *a* > 29% germline transmission rate (Table 1). Interestingly, six out of these eight pairs showed correct integration at both the 5′ and 3′ junctions, resulting in a 21% germline transmission rate of the correct knock-in allele. By out-crossing these 12 fish with wild-type fish, one fish from each pair (either the male or the female) was identified as a founder. Out-crossing of FLAGx3-50_#23 fish suggested germline mosaicism, as demonstrated by a mix of trace data for the 5′ junction amplifications (Supplementary Figure 1Ba).

**TABLE 1:** Summary of germline transmission of knocked-in *sox3* alleles.

Tag-3′ homology arm length	Number of fish screened	Number of germline transmitted fish (F0 founders)	Rate of germline transmission	F0 founders with correct integration		Rate of F1 embryos with correct integration
					
				Rate	ID #	
FLAGx3-300	37	7	19% (7/37)	3% (1/37)	#9	16% (8/50)
FLAGx3-50	28	8	29% (8/28)	21% (6/28)	#9	ND
					#16	85% (17/20)
					#19	5% (1/20)
					#20	ND
					#21	50% (12/24)
					#22	10% (2/20)
PAx3-300	30	5	17% (5/30)	3% (1/30)	#7	ND
PAx3-50	47	10	21% (10/47)	6% (3/47)	#21	55% (11/20)
					#25	ND
					#34	ND

*ND, not determined.*

Similarly, we examined the effect of using the lssDNA donor template with either the 300-nt or 50-nt 3′ homology arm on the germline transmission rates of PAx3 composite tag knock-ins. First, 30 F0 fish that had been injected with the 300-nt 3′ homology arm lssDNA donor template were screened; five in-crossed pairs were positive for knock-in allele-specific PCRs, with *a* > 17% germline transmission rate. One pair exhibited a correct integration at both the 5′ and 3′ junctions; however, when out-crossed with the wild-type fish, neither fish produced embryos positive for the tag integration, suggesting a very low percentage of knock-in allele positive germ cells. Afterward, 47 F0 fish injected with the lssDNA donor template with a 50-nt 3′ homology arm were screened. Ten in-crossed pairs were positive for knock-in allele-specific PCRs, resulting in *a* > 21% germline transmission rate. Three pairs out of 10 exhibited correct integration at both the 5′ and 3′ junctions, and by out-crossing these six fish with wild-type fish, either a male or a female of each pair was identified as a founder.

For all four scenarios analyzed herein, the germline transmission rates of either the FLAGx3 or PAx3 composite tag knock-ins were high (17–29%) when we included the imprecisely edited alleles. Among them, the germline transmission rates using the lssDNA donor templates with a 50-nt 3′ homology arm were higher than those with a 300-nt 3′ homology arm (Table 1). Moreover, this tendency was more pronounced for the correct integration rates, with the shorter homology arm being substantially more effective than the longer one (Table 1). Regardless of homology arm length, the majority of the imprecisely repaired sequences were found to have occurred at the 5′ junction, some of which appeared to be associated with large deletions given that no PCR amplicons were obtained at this junction (Table 1 and Supplementary Figure 1). These results are somewhat consistent with those of the knock-in allele-specific qPCR assays using the embryos injected with PAx3 composite tag knock-in donors (Figure 5C).

**FIGURE 6:**
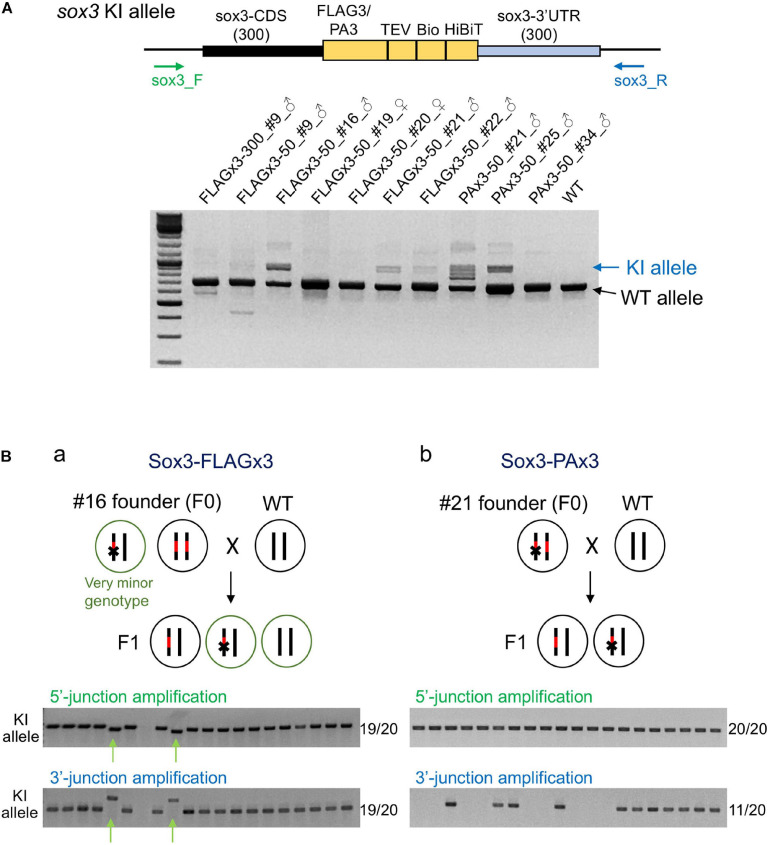
Founder germ cell mosaicism. **(A)** Mosaicism analysis by PCR using genomic DNA from F1 embryo pools. F1 embryos from out-crosses of the founders with wild-type fish were pooled (50–100 embryos per pool) and used for genomic DNA preparation. The PCR primers bind outside of the homology arms to amplify both wild-type (WT) and knock-in (KI) alleles as indicated by the arrows. The agarose gel electrophoresis image represents the PCR amplicons of each founder along with the wild-type fish. **(B)** Mosaicism analysis by PCR using genomic DNA from individual F1 embryos. Possible genotypes of germ cells of biallelic knock-in founders and F1 embryos are illustrated. Individual F1 progeny embryos from out-crosses of FLAGx3-50_#16 **(a)** and PAx3-50_#21 **(b)** founders were subjected to genomic DNA preparation and PCR amplification of 5′ and 3′ junctions of the composite tag-modified *sox3* gene. A total of 20 embryos were analyzed per founder. The number of PCR positive embryos per total embryos for each PCR amplification is shown on the right side of each agarose gel image. The minor knock-in allele with imprecisions is indicated with green arrows **(Ba)**.

To determine the abundance of knock-in-allele-positive germ cells, the founder fish were further out-crossed with wild-type fish, and pooled F1 embryos were analyzed *via* PCR using primers that bind outside the homology arms to simultaneously amplify the knock-in and wild-type alleles. In these PCR assays, the wild-type allele was preferentially amplified due to its shorter length compared with the knock-in allele. Amplification of the knock-in allele exhibited varying intensities, suggesting that the abundance of knock-in allele positive germ cells varied among the founders (Figure 6A). Notably, some founders exhibited a strong PCR signal for the knock-in allele, suggesting that biallelic insertion may have occurred in the primordial germ cells at the early embryonic stage. Therefore, we performed knock-in allele-specific PCR at both the 5′ and 3′ junctions for individual F1 embryos to examine germline mosaicism. Interestingly, for the FLAGx3-50_#16 founder, 17 embryos out of 20 rendered the correct PCR amplicons at both 5′ and 3′ junctions, suggesting that the majority of the germ cells of this founder were homozygous for the composite tag insertion. The remaining two embryos showed different lengths for both PCR products, suggesting the occurrence of multiple knock-in events, at least one of which was a minor event. The genotype of these minor germ cells was likely heterozygous for the incorrect integration (Figure 6Ba). The PAx3-50_#21 founder also exhibited a biallelic behavior for the knock-in, as evidenced by a positive PCR amplification at the 5′ junction in all tested embryos. Roughly half of the embryos were positive for the PCR amplification of the 3′ junction, suggesting that one knock-in allele was associated with a large deletion (Figure 6Bb). For the other founders, varying rates of F1 embryos with knock-in alleles were observed (Table 1), which is consistent with the band intensities of the knock-in allele PCR products using the pooled samples (Figure 6A). These results collectively indicate that composite tag knock-ins could be accurately and efficiently achieved using lssDNA donor templates in conjunction with the CRISPR-Cas9 RNP complex.

### Validation of the Tagged Sox3 Protein Expression

**FIGURE 7:**
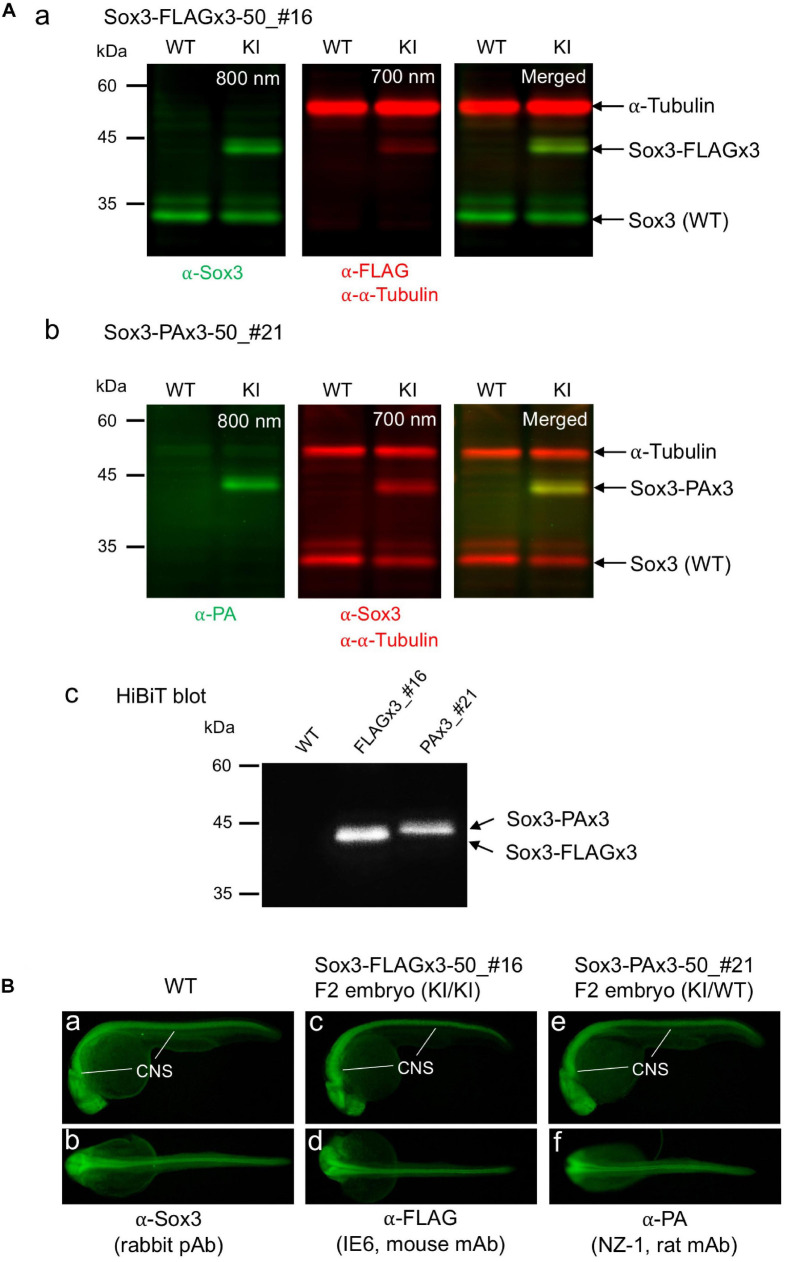
Expression of the tagged Sox3 protein. **(A)** Western blot and HiBiT blot analyses of the tagged Sox3 protein. Two-color Western blot analysis of *sox3* knock-in embryos derived from the FLAGx3-50_#16 **(a)** and PAx3-50_#21 founders **(b)** with antibodies against Sox3, FLAG tag or PA tag, and α-tubulin. HiBiT blotting using an excessive amount of LgBiT protein and substrate to detect the C-terminal HiBiT peptide of the tagged Sox3 proteins **(c)**. **(B)** Whole-mount immunohistochemical staining for the tagged Sox3 protein. Each primary antibody used to stain zebrafish is indicated below each image. Genotyping was conducted using PCR with genomic DNA prepared from the immunostained embryos after image acquisition.

To confirm that the endogenously tagged Sox3 protein expression was not adversely affected by knock-in, Western blotting and HiBiT blotting were performed using F1 embryos derived from the founders with biallelic insertions (FLAGx3-50_#16 and PAx3-50_#21). As expected, using either anti-FLAG or anti-PA antibodies in addition to anti-Sox3 antibodies, the tagged Sox3 proteins were detected with their expected molecular sizes on the blot (Figures 7Aa,b), indicating that the composite tags did not affect the expression and stability of the Sox3 protein. Moreover, in both cases, the tagged Sox3 proteins were also detected by HiBiT blotting (i.e., a luminescence detection method based on a C-terminus HiBiT tag) (Figure 7Ac), which confirmed that the tagged Sox3 proteins remained intact.

By crossing the heterozygous F1 knock-in fish derived from either the FLAGx3-50_#16 or PAx3-50_#21 founders, F2 embryos were obtained at near Mendelian ratios (Supplementary Figure 2), suggesting that these knock-in alleles were not deleterious to development. Using these F2 embryos, the expression of these tagged Sox3 proteins was further validated *via* whole-mount immunohistochemical analysis. Consistent with the blotting data, the expression of the Sox3 protein tagged with either the FLAGx3 or PAx3 composite tag was detected with anti-FLAG or anti-PA antibodies in the central nervous system in essentially the same pattern as that of wild-type Sox3 stained with anti-Sox3 antibodies (Figure 7B), which further confirmed that the tagged Sox3 proteins were normally expressed.

### Optimal lssDNA Structure for Efficient Knock-Ins

**FIGURE 8:**
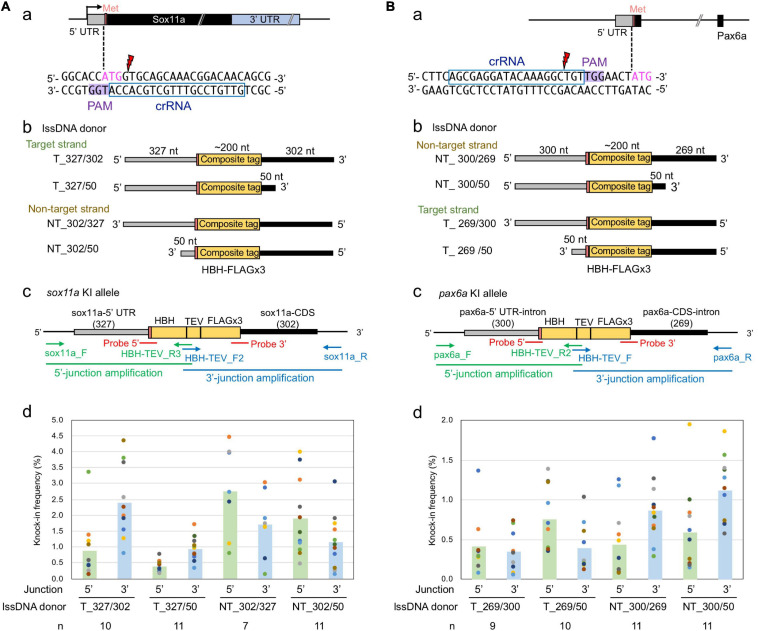
Effect of lssDNA strand choice and 3′ homology arm length on composite tag knock-in into the *sox11a* and *pax6a* genes. In these knock-in designs, ∼200-nt-long composites that contain the HBH (His6-Bio-His6) tag followed by a TEV protease cleavage site and FLAG epitope tag in its trimeric form were knocked into the 5′ end of the coding sequence of the *sox11a* **(A)** and *pax6a* **(B)** genes. **(a)** Sequences and locations of crRNAs for DSB induction of *sox11a* and *pax6a*. **(b)** Target and non-target strands of lssDNA with different 3′ homology arm lengths were used as donor templates. Each lssDNA was microinjected with 1.5 fmol of the RNP complex into one-cell stage zebrafish embryos, and genomic DNA was extracted from 10-embryo pools. **(c)** Schematic illustration of the *sox11a* and *pax6a* knock-in alleles and knock-in allele-specific PCRs. **(d)** Knock-in allele-specific qPCRs for 5′ and 3′ junctions using the hydrolysis probes shown in panel **(c)**. The vertical bars represent the means of 7–11 replicates, each of which consists of a pooled sample of 10 injected embryos and is shown as a colored circle.

The above-described *sox3* gene-targeted knock-in experiments demonstrated that strand selection and 3′ homology arm length were critical parameters for optimal lssDNA donor template design. Therefore, we further explored whether these two parameters would remain important when designing knock-in donor templates for other gene loci. For this purpose, the zebrafish *sox11a* and *pax6a* genes were selected as knock-in targets because efficient crRNAs were successfully identified in the immediate vicinity of the intended editing sites (Figures 8Aa,Ba). Cleavage efficiencies of CRISPR-Cas9 RNPs containing these crRNAs were analyzed with the ICE tool as described for *sox3*. When 1.5 fmol of RNP complex was used for injection, frequencies of indel occurrence were 86% for *sox11a* and 70% for *pax6a*. In these knock-in experiments, we aimed to accurately knock-in an ∼200-nt-long composite tag sequence that encodes the HBH tag (Bio tag flanked by two hexahistidine tags) and the FLAGx3 tag (Supplementary Figure 3) at the 5′ end of the coding sequence of *sox11a* or *pax6a*.

To examine the effect of these donor template design parameters on knock-in efficiency, four lssDNA donor templates were created for each target with varying strand choices and 3′ homology arm lengths (Figures 8Ab,Bb). These lssDNA donors were microinjected into approximately 100 one-cell stage embryos along with the respective CRISPR-Cas9 RNP complex, and genomic DNA was prepared from 10-embryo pools at approximately 24–26 hpf, after which knock-in allele-specific qPCRs for the 5′ and 3′ junctions were then performed in a similar way as the *sox3* knock-in qPCR experiments (Figures 8Ac,Bc). Interestingly, strand preference was still observed for both the *sox11a* and *pax6a* targets, where the non-target strand appeared to be more efficient in both knock-ins. However, the differences were smaller than those observed for the *sox3* knock-in. Regarding 3′ homology arm length, the lssDNA donor templates with the shorter 50-nt arm rendered slightly higher knock-in efficiencies for the *pax6a* knock-in, which was similar to the *sox3* knock-in. In contrast, for the *sox11a* knock-in, the lssDNA donor templates with the 300-nt arm resulted in slightly higher knock-in efficiencies. Taken together, these results suggest that strand preference and optimal 3′ homology arm length are dependent on the target gene or its sequence.

As observed for the *sox3* knock-in, the knock-in allele frequencies were higher at the 3′ side of the lssDNA donors in all the tested cases (i.e., the 3′ junction for the target strand donors and 5′ junction for the non-target strand donors of the *sox11a* knock-in; and the reversed relation for the *pax6a* knock-in donors). Thus, the higher frequencies of precisely edited knock-in alleles at the 3′ side of the ssDNA donors may be universal for ssDNA-mediated knock-in, where the repair process is thought to be initiated from the 3′ side (see the section “Discussion”).

### Germline Transmission of lssDNA-Mediated Knocked-in *pax6a* and *sox11a* Alleles

To further explore the knock-in efficiency of lssDNA donor templates in germline cells, the four *pax6a* lssDNA donor templates (Figure 8Bb) were each injected into the zebrafish embryos along with the CRISPR-Cas9 RNP complex. Similar to the *sox3* knock-in embryos, the injected embryos were then raised to adulthood and tested for germline transmission. Ten to 36 F0 fish were screened for each lssDNA donor, and at least 17 fish were positive for knock-in allele-specific PCRs among a total of 79 F0 fish; this corresponded to *a* > 22% germline transmission rate on average (Table 2). Upon comparing the germline transmission rates among the four lssDNA donor templates, the target strand donor with the longer 3′ homology arm (T_269/300) exhibited the lowest rate, which appeared to roughly correlate with the frequencies of the knock-in alleles determined using the injected embryos and qPCR (Figure 8Bc). Although the germline transmission rates for the *pax6a* knock-in exhibited similar ranges to those obtained for the *sox3* knock-in, only the three F0 fish transmitted the correctly edited knock-in allele (Table 2 and Supplementary Figure 4). This lower transmission rate may have been due to the distance between the CRISPR-Cas9 cleavage site and the intended tag insertion site (10 nt for the *pax6a* knock-in versus 2 nt for the *sox3* knock-in) (Figure 8Ba). This is supported by the reported monotonic inverse relationship between the rate of point mutation incorporation and distance from the cleavage site, where the rate decreases to approximately 50% with 10 nt (Paquet et al., 2016). Additionally, the DNA sequence characteristics of the *pax6a* locus might adversely affect the repair process. For example, there is a homopolymeric sequence (TTTTT/AAAAA) that occurs repeatedly in the *pax6a* homology arm sequence.

**TABLE 2:** Summary of germline transmission of knocked-in *pax6a* alleles.

Strand_5′–3′ homology arm length	Number of fish screened	Number of germline transmitted fish (F0 founders)	Rate of germline transmission	F0 founders with correct integration		Rate of F1 embryos with correct integration
					
				Rate	ID #	
T_269/300	21	3	14% (3/21)	5% (1/21)	#6	17% (4/24)
T_269/50	22	5	23% (5/22)	5% (1/22)	#5	2% (1/44)
NT_300/269	10	2	20% (2/10)	0% (0/10)		
NT_300/50	36	7	20% (7/36)	3% (1/36)	#2–3	13% (3/23)

To assess the germline transmission efficiency for the *sox11a* knock-in, we injected the *sox11a* lssDNA donor template T_327/302 that contains the approximately 300-nt homology arms on both sides (Figure 8Ab) along with the CRISPR-Cas9 RNP complex into embryos and raised them to adulthood. Twenty-five F0 fish were screened, and eight fish were found to be positive in the knock-in allele-specific PCRs, resulting in a 32% germline transmission rate (Table 3 and Supplementary Figure 5). Among them, three F0 fish transmitted the precisely edited knock-in allele to F1 embryos (Table 3). This relatively high success rate may also be explained by the distance between the CRISPR-Cas9 cleavage site and the intended tag insertion site (4 nt for the *sox11a* knock-in) (Figure 8Aa).

**TABLE 3:** Summary of germline transmission of knocked-in *sox11a* alleles.

Strand_5′–3′ homology arm length	Number of fish screened	Number of germline transmitted fish (F0 founders)	Rate of germline transmission	F0 founders with correct integration		Rate of F1 embryos with correct integration
					
				Rate	ID #	
T_327/302	25	8	32% (8/25)	12% (3/25)	#1	25% (5/20)
					#2	ND
					#11	13% (4/31)

*ND, not determined.*

Taken together, these results suggest that ∼200 base-pair sequence encoding a composite tag can be efficiently knocked-in into the zebrafish genome using a combination of the CRISPR-Cas9 RNP complex and a lssDNA as a donor template; however, the correct knock-in success rate could vary depending on several important factors including lssDNA strand selection, homology arm length, distance from the DSB to the knock-in site, and the characteristics of the target genome sequences.

## Discussion

Precise genome editing using CRISPR-Cas9-mediated knock-ins in zebrafish remains an important challenge and therefore requires further efficiency and precision optimization. In this study, several parameters were evaluated to improve the efficiency of ∼200-nt-long composite tag sequence knock-ins. Importantly, our study identified three factors that affect knock-in efficiency. First, using lssDNA as a donor template coupled with the CRISPR-Cas9 RNP complex yielded efficient composite tag knock-ins. Second, a shorter (i.e., 50-nt) 3′ homology arm enhanced knock-in efficiency compared with longer arms in a knock-in site-dependent manner. Third, efficiency also depends on strand choice; however, target or non-target strand preference appears to depend on the target locus and/or its sequence. Finally, our approach successfully rendered the precisely edited *sox3*, *sox11a*, and *pax6a* knock-in alleles containing a composite tag composed of FLAGx3/PAx3-Bio-HiBiT or His-Bio-His-FLAGx3 with high to moderate germline transmission rates.

In zebrafish, initial attempts to insert short sequences, such as restriction enzyme sites (Bedell et al., 2012), LoxP sites (Bedell et al., 2012; Chang et al., 2013), epitope tags (Hruscha et al., 2013), and single nucleotide substitutions (Hwang et al., 2013), have utilized chemically synthesized ssODNs due to the moderate costs associated with implementing this technology, provided that the required ssODNs are <200 bases in length. Recent advances in lssDNA synthesis methods using nicking enzymes (Yoshimi et al., 2016; Miyasaka et al., 2018) or Easi-CRISPR (Quadros et al., 2017; Miura et al., 2018) enable the insertion of longer sequences. However, despite being more cost-effective than chemical lssDNA synthesis, these approaches have not been widely adopted likely due to their labor-intensive implementation. With the exception of a recent study by Bai et al. (2020), lssDNAs have otherwise not been applied in zebrafish knock-in studies. We found that lssDNA donors elicited a higher knock-in efficiency with lower embryo toxicity than dsDNA donors, suggesting that lssDNA could be especially well-suited for knock-in of relatively long tags, such as multimerized epitope tags and composite tags. An additional advantage of lssDNA-mediated knock-in is that it exhibits low levels of homology-independent off-target integration, which is often associated with dsDNA-mediated knock-ins (Roth et al., 2018; Li et al., 2019). Our data indicated that strand selection is critical to achieving efficient knock-in using lssDNA donors, as evidenced in the *sox3* knock-in; however, this selection appears to be locus- or sequence-specific, which is consistent with previous reports using short ssDNA donors (Hwang et al., 2013). A recent study showed that lssDNA donor templates are also well-suited for the introduction of point mutations and short sequence insertions in zebrafish (Bai et al., 2020). Nonetheless, it is currently unclear whether the proposed lssDNA knock-in strategy is also suitable for longer insertions (e.g., fluorescent protein-coding sequences) in zebrafish, as our preliminary experiments demonstrated that a lssDNA donor for 2A-EGFP insertion into the *sox3* gene did not yield a satisfactory knock-in efficiency. For such knock-in applications, short homologous sequence-mediated knock-in methods (e.g., GeneWeld and PITCh systems) may be advantageous, where short homologous ends between the genomic target site and donor fragment are generated by their simultaneous DNA cleavage by CRISPR-Cas9 (Hisano et al., 2015; Luo et al., 2018; Wierson et al., 2020).

Our qPCR-based assessment demonstrated that the use of 50-nt 3′ homology arms resulted in slightly higher knock-in efficiency than that of ∼300-nt arms in the somatic cells of injected embryos for the *sox3* and *pax6a* knock-in experiments, whereas the opposite was observed for the *sox11a* knock-in. Additionally, we observed that implementing 50-nt 3′ homology arms resulted in more efficient and accurate knock-ins of germline-transmitted *sox3* gene alleles. DSB repair with ssDNA is thought to be mediated by a process known as SSTR, which is known to be independent of the Rad51 strand exchange protein (Jasin and Haber, 2016; Gallagher and Haber, 2018). During the SSTR repair of a DSB, resection of the 5′ ends of the DSB creates 3′ overhangs, and the 3′ end of the ssDNA donor pairs with one of the overhangs, followed then by DNA synthesis to copy the remaining ssDNA donor sequence. In this ssDNA-driven repair model, shorter 3′ homology arms are thought to facilitate the binding of the donor template to the protruding single-stranded 3′ regions, which is consistent with the findings of a short ssDNA donor study that explored the introduction of point mutations, where ssDNA donors with 30–36 nt 3′ homology arms performed efficiently (Richardson et al., 2016; Liang et al., 2017; Boel et al., 2018). However, our qPCR data on the *sox11a* knock-in suggest that the optimal length of the 3′ homology is dependent on the nature of the knock-in sites and thus may need to be empirically determined at least for long ssDNAs. According to the SSTR repair model, the final process involves the displacement of the extended strand from the template strand, followed by reannealing with the single-stranded tail on the other DSB end. This proposed unidirectional repair process is consistent with our qPCR data, in which the frequencies of knock-in alleles were higher at the 3′ side of the lssDNA donors in all the tested lssDNA templates for the three knock-in targets, suggesting that the lssDNA-templated knock-in is actually mediated by the SSTR pathway in zebrafish embryos. Furthermore, the length of the 5′ homology arm may affect the final repair process. Our study implemented ∼300-nt-long 5′ homology arms to avoid the potential exonuclease activity, as suggested by Yoshimi et al. (2016). However, given that we still observed high rates of erroneous repair events at the 5′ position in the germline transmitted alleles, further studies will be required to determine an optimal 5′ homology arm length.

The germline transmission data derived from the *sox3*, *sox11a*, and *pax6a* knock-in experiments demonstrate that the composite tag knock-ins themselves occurred at high rates (i.e., approximately 20%) in every setting when we counted both the correct and incorrect alleles. However, the germline transmission rates of the precisely edited alleles varied considerably among them. Several factors could account for this difference. One important factor could be the distance between the CRISPR-Cas9 cleavage site and the intended insertion site, as a stereotyped inverse relationship was reported between the base incorporation rate by ssDNA-mediated editing and its distance from the CRISPR-Cas9 cleavage site (Paquet et al., 2016). Additionally, some DNA sequence characteristics of the knock-in donors and/or target site sequences appear to adversely affect the repair process, leading to editing errors. In the case of the *sox3* knock-ins, the FLAGx3 composite tag knock-in donor with the 50-nt 3′ homology arm resulted in more efficient germline transmission of the precisely edited allele than that of PAx3 despite having the same sequence except for the epitope tag-encoding sequences. Some characteristics of PA tag-encoding sequences [e.g., higher GC-content (63.2% for PAx3 versus 38.5% for FLAGx3) and/or secondary structure] may increase the frequency of erroneous repair events. In the case of the *pax6a* knock-ins, the frequent occurrence of low complexity sequences, such as a homopolymeric sequence (TTTTT/AAAAA) in the *pax6a* homology arm sequence, might prevent the correct lssDNA-mediated repair. The repetitive nature of multimerized tags (three copies of FLAG and PA tags, two copies of the His tag) could also result in erroneous repair events. In fact, a recent study proposed that the presence of microhomology may cause erroneous repair in ssDNA-mediated genome editing (Boel et al., 2018). These short homologous sequences might have caused partial deletion and duplication, as we often observed deletions in the FLAGx3 sequence, particularly for the *pax6a* knock-in (Supplementary Figure 4). However, we have not identified any evidence of multiple head-to-tail insertions of the donor templates, although such events were observed for mouse knock-in experiments even with lssDNA donors (Skryabin et al., 2020).

In our knock-in study, we employed CRISPR-Cas9 RNPs that had been assembled *in vitro* using two synthetic RNA oligonucleotides (a target-specific crRNA and a universal tracrRNA) and the Cas9 protein to introduce DSBs. Recent reports demonstrated that the same crRNA/tracrRNA-based RNP system is highly mutagenic in zebrafish embryos (Hoshijima et al., 2019; DiNapoli et al., 2020). RNP-injected embryos often show null mutant phenotypes (Hoshijima et al., 2019). Thus, the high knock-in efficiency obtained herein may be partly attributable to the use of the crRNA/tracrRNA-based RNP system; however, direct comparisons with single-guide systems are yet to be conducted to support this hypothesis.

By using lssDNA donor templates coupled with a crRNA/tracrRNA-based RNP system, we successfully obtained precisely edited knock-in alleles with variable germline transmission rates. Specifically, 21% of the screened fish transmitted an accurately edited composite tag-containing allele in the best-case scenario of the *sox3* knock-in. The germline transmission rates of the precisely edited alleles obtained in this study (3–21%) were similar to or higher than those reported in previous zebrafish knock-in studies that employed short ssDNA templates to introduce point mutations and epitope tag insertions, where pre-screening of injected embryos with fluorescent signals, for instance, was not applicable due to the nature of the insertions. Armstrong et al. (2016) reported germline transmission rates of 2–4% for point mutations in the zebrafish *tardbp* and *fus* genes, which corresponded with mutations observed in amyotrophic lateral sclerosis (ALS) patients. Regarding epitope tag knock-ins, although the insert length of a monomeric V5 tag in a previous study was relatively shorter than our composite tags, the knock-in experiment to insert the V5 tag into the *tcf21* and *tbx18* genes using ssODN templates with nearly 20-nt-long homology arms respectively resulted in germline transmission rates of 0 and 12.5% of the accurately edited tag integration (Burg et al., 2016). A recent study that used lssDNAs for the introduction of point mutations reported efficient germline transmission of knock-in alleles (9.5–31.8%) (Bai et al., 2020). Therefore, high rates of accurate and heritable integrations appear to be achieved using lssDNA knock-in donors aimed at introducing point mutations to composite tags. However, further evidence of efficient integration for other genes/loci is still needed.

## Materials and Methods

### CRISPR-Cas9 RNP Complex Microinjection Into Zebrafish Embryos

CRISPR-Cas9 crRNAs were designed using the IDT CRISPR design tool. The crRNA sequences are illustrated in Figures 2, 8. The CRISPR-Cas9 RNAs (i.e., crRNA and tracrRNA) and the Cas9 protein (Alt-R S.p. HiFi Cas9 Nuclease V3) were purchased from IDT, and the CRISPR-Cas9 RNP complex was assembled according to the manufacturer’s protocol for zebrafish embryo microinjections. In brief, to create a 3 μM gRNA complex solution, equimolar amounts of crRNA and tracrRNA were mixed in a nuclease-free duplex buffer (IDT). The solution was heated at 95°C for 5 min and subsequently cooled to room temperature. The gRNA complex solution was then mixed with an equal volume of 3 μM Cas9 protein solution diluted in Cas9 working buffer (20 mM HEPES–KOH, 150 mM KCl, pH 7.5), after which the mixture was incubated at 37°C for 10 min to assemble the RNP complex at 1.5 μM. To assess the cleavage efficiency of CRISPR-Cas9, 1 or 2 nl of the RNP complex solution was microinjected into one-cell stage TL zebrafish embryos, resulting in a 1.5 or 3 fmol RNP complex delivery.

For knock-in experiments, the prepared lssDNA solution was mixed with the 1.5 μM RNP complex to obtain a final concentration of 0.2 μM lssDNA and 1 μM RNP complex in the injection solution. A 1.5 nl volume of the lssDNA/RNP solution was then microinjected into one-cell stage TL zebrafish embryos, resulting in a final delivery of 0.3 fmol of lssDNA and 1.5 fmol of the RNP complex per embryo. The lssDNA/RNP solution had been routinely injected into the cytoplasm of one-cell stage embryos to ensure the early delivery of the lssDNA donors to the genomic target site.

To compare donor DNA template types, 75 pg (0.3 fmol) of lssDNA, 75 pg (0.15 fmol) of PCR fragments, or 75 pg (0.034 fmol) of plasmid DNA (pUC19-[sox3-CDS300]-FLAGx3-TEV-Bio-HiBiT-[3′ UTR300]) was microinjected with 1.5 fmol of the RNP complex into the cytoplasm of one-cell stage zebrafish embryos, after which genomic DNA was extracted from 20 individual zebrafish embryos.

To assess cleavage and knock-in efficiency, genomic DNA was prepared from single embryos or pools of normally developing 1 dpf embryos using genomic DNA extraction buffer [200 mM NaCl, 10 mM Tris–HCl (pH 8), 10 mM EDTA, 1% Triton X-100, 200 μg/ml Proteinase K] or low-EDTA genomic DNA extraction buffer [10 mM Tris–HCl (pH 8), 0.1 mM EDTA, 0.2% Triton X-100, 200 μg/ml Proteinase K]. The embryos were incubated at 55°C for 2–3 h with occasional mixing until they dissolved completely. Proteinase K was inactivated by heating the samples at 90°C for 10–12 min. This crude genomic DNA solution was directly used in PCR assays.

### Heteroduplex Mobility Assay

Heteroduplex mobility assay primers were designed to amplify an approximately 150-bp region surrounding the stop codon of the *sox3* gene (primer sequences are listed in Supplementary Table 1). PCRs were performed with Taq DNA polymerase (New England BioLabs) under the following conditions: 95°C for 30 s; 30 cycles of 95°C for 15 s, 55°C for 30 s, and 68°C for 10 s; and 68°C for 5 min, followed by denaturation for 5 min at 95°C. The resulting PCR products were then removed from the thermocycler and maintained at room temperature for at least 5 min to allow for annealing, after which they were loaded onto a non-denaturing polyacrylamide gel containing 15% acrylamide–bisacrylamide (29:1) in 1 × Tris–borate–EDTA (TBE) buffer. After electrophoresis, the polyacrylamide gel was immersed in a 0.5 μg/ml ethidium bromide solution for 40 min and then visualized in a Fusion imaging system (Vilber-Lourmat).

### Plasmid Construction for Donor Templates

We first cloned the fragments of TL zebrafish genomic DNA that encompass the knock-in sites of the *sox3*, *sox11a*, and *pax6a* genes to verify their genomic sequences. DNA fragments of the left and right homology arms of the donor DNA template were amplified using specific primers (the primer sequences are listed in Supplementary Table 1). These 5′ and 3′ arm fragments were cloned into a pUC19 vector along with a DNA fragment encoding the composite tag to obtain the donor plasmid (i.e., pUC19-[sox3-CDS300]-FLAGx3-TEV-Bio-HiBiT-[3′ UTR300], etc.). The details of the FLAGx3-Bio-HiBiT and PAx3-Bio-HiBiT composite tags were described in our previous study (Ranawakage et al., 2019). The HBH-FLAGx3 composite tag contains a biotin acceptor domain (Bio-tag) flanked by 6xHis, a tobacco etch virus (TEV) protease cleavage site, and FLAG in its trimeric form (the exact nucleotide and amino acid sequences of the HBH-FLAGx3 tag are summarized in Supplementary Figure 3). The *sox3* plasmid construct was used as the plasmid knock-in donor and template to generate the dsDNA knock-in donor *via* PCR.

### Long ssDNA Preparation

lssDNAs were prepared using the LsODN preparation kit (Biodynamics Laboratory, Japan) according to the manufacturer’s instructions. The donor fragments in pUC19 described above were sub-cloned into the *Eco*RV or *Sma*I site of the pLSODN-1 plasmid. The resulting plasmids were digested with nicking and restriction endonucleases: Nt.BspQI and Nb.*Bbv*CI for *sox3*-HA_FLAGx3-Bio-HiBiT, *Hin*dIII and Nt.BspQI for *sox3*-HA_PAx3-Bio-HiBiT, Nt.BspQI and Nb.*Bsr*DI for *sox11a*-HA_HBH-FLAGx3, Nt.BspQI and Nb.*Bbv*CI for *pax6a*-HA (target 269/300, 269/50)_HBH-FLAGx3, and Nt.*Bbv*CI and *Pst*I for *pax6a* (non-target 300/269, 300/50)-HA_HBH-FLAGx3. The nicked plasmids were incubated with denaturing gel loading buffer (Biodynamics Laboratory) and subjected to agarose gel electrophoresis. After electrophoresis, the gel was stained with a 0.5 μg/ml ethidium bromide solution. The bands corresponding to lssDNA donor fragments were excised and extracted using NucleoSpin Gel and PCR Clean-up columns (MACHEREY-NAGEL).

### Knock-in Efficiency Evaluation and F0 Fish Screening via PCR

Polymerase chain reaction was performed using the primer pairs listed in Supplementary Table 1. Each 30-μl PCR reaction mixture contained 0.75 U of Taq DNA Polymerase (New England BioLabs), 1 × ThermoPol buffer, 0.5 μM forward and reverse primer pairs, 0.2 mM dNTP mix, 1 × sucrose red solution (10% sucrose, 0.17 mM cresol red), and genomic DNA template (1–2 μl of the embryo lysate). The standard PCR conditions were the following: 95°C for 30 s; 30 cycles of 95°C for 15 s, 55–62°C for 30 s, and 68°C for 1 min/kb; and 68°C for 5 min.

Knock-in allele-specific qPCR was performed using the primer pairs and hydrolysis probes listed in Supplementary Table 2 using the Hot Start TTx (DNA) Kit (Toyobo). qPCR for the *hesx1* promoter region was performed with Luna Universal Probe qPCR Master Mix (New England BioLabs) and was used for normalization. The probes were labeled at the 5′ end with FAM dye, and a combination of Zen and Iowa black FQ quenchers was used to label the 3′ end (IDT). Each 20-μl qPCR reaction contained 0.4 μM forward and reverse primers, 0.2 μM probe, and genomic DNA template (2 and 1 μl of the embryo lysate for the knock-in allele-specific and *hesx1* qPCRs, respectively). The amplification reaction consisted of an initial denaturation step at 95°C for 1 min, followed by 45 cycles of 95°C for 15 s and 60–64°C for 60 s (Supplementary Table 2 for annealing/extension temperatures). The reactions were monitored with a LightCycler 96 Real-Time PCR System (Roche), and the data were analyzed using the software provided by the manufacturers. For copy number quantitation of knock-in alleles, genomic DNA templates were spiked with serial dilutions of linearized plasmids containing the knock-in alleles. The copy numbers of the zebrafish genome were assessed by *hesx1* qPCR using concentration-adjusted genomic DNA as a standard. The knock-in allele frequency was then obtained by dividing the knock-in alleles copy number by the genomic DNA copy number.

### Whole-Mount Immunohistochemistry

Whole-mount immunohistochemistry was performed as described by Inoue and Wittbrodt (2011) with slight modifications. The heterozygous F1 knock-in male and female fish derived from the FLAGx3-50_#16 or PAx3-50_#21 founders were crossed to obtain the respective F2 embryos. These F2 embryos were then dechorionated at 24 hpf, fixed in freshly prepared 4% formaldehyde/PBS for 2 h at 4°C, and washed three times with PBST (5 min per wash at room temperature). Afterward, the embryos were dehydrated by incubating them in successive methanol dilutions in PBST: 25, 50, 75, and 100% (v/v) methanol (5 min incubations per dilution). The embryos were then rehydrated in the opposite order with the same methanol series and washed three times with PBST (5 min per wash at room temperature). Afterward, the embryos were washed with 150 mM Tris–HCl (pH 9.0) for 5 min and incubated with 150 mM Tris–HCl (pH 9.0) for 15 min at 70°C for antigen retrieval, followed by three 5-min PBST washes. On an ice bath, the embryos were rinsed twice with ice-cold H_2_O and permeabilized in prechilled acetone for 20 min at -20°C. The embryos were then rinsed twice with ice-cold H_2_O to remove the acetone. Next, the embryos were washed twice with PBST (5 min per wash at room temperature) and blocked with a blocking buffer {10% normal goat serum in PBT [0.1 M sodium phosphate buffer (pH 7.4), 0.8% Triton X-100]} at 4°C for 3 h. The embryos were then transferred into a 24-well plate and incubated in 1% normal goat serum/PBT with a primary antibody for 3 days at 4°C with rocking agitation. The primary antibodies used were anti-Sox3 (rabbit pAb, GTX132494, GeneTex) at 5 μg/ml, anti-FLAG (mouse mAb, IE6, Wako) at 10 μg/ml, and anti-PA (rat mAb, NZ-1, Wako) at 10 μg/ml. Afterward, the embryos were washed five times with PBT (1 h per wash at room temperature) and subsequently incubated in 1% normal goat serum/PBT with a secondary antibody (anti-rabbit IgG Alexa 488, anti-mouse IgG Alexa 488, or anti-rat IgG Alexa 488) at a final concentration of 4 μg/ml for 2 days at 4°C with rocking agitation, followed by five PBT washes (5 min per wash at room temperature). Finally, the embryos were mounted with a glycerol gradient in PBS [25, 50, and 75% (vol/vol) glycerol; 20 min in each glycerol dilution], and the staining was visualized under a fluorescent microscope.

### Western Blotting and HiBiT Blotting

Western blotting and HiBiT blotting were performed as described in our previous study (Ranawakage et al., 2019). F1 progeny embryos were collected by out-crossing the Sox3-FLAGx3-KI_#16 and PAx3-KI_#21 F0 founders with wild-type fish and reared to the 70–80% epiboly stage for protein preparation. To simultaneously detect Sox3, α-tubulin, and FLAG or PA tags using two-color fluorescent Western blots, the membranes were probed with anti-Sox3 (0.5 μg/ml, GTX132494; GeneTex), anti-α-tubulin (1 μg/ml, B-5-1-2; Sigma), and each of anti-FLAG (0.5 μg/ml, M2; Sigma) or anti-PA (0.5 μg/ml, NZ-1; Wako) antibodies; the antibodies were diluted in Can Get Signal solution 1 (Toyobo). For the Sox3-FLAGx3-KI_#16 membrane, the Sox3 antibody was detected with goat anti-rabbit IgG-IRDye800, and the α-tubulin and FLAG antibodies were detected with goat anti-mouse IgG-IRDye680. For the Sox3-PAx3-KI_#21 membrane, the Sox3 antibody was detected with goat anti-rabbit IgG-CF680, the α-tubulin antibody with goat anti-mouse IgG-IRDye680, and the PA antibody with goat anti-rat IgG-IRDye800. All secondary antibodies were diluted in Odyssey blocking buffer (1:1 diluted with TBS) containing 0.1% Tween-20 and 0.01% SDS. Images were acquired in both 700 and 800 nm fluorescent channels using an Odyssey CLx infrared imaging system (LI-COR Biosciences).

## Data Availability Statement

The original contributions presented in the study are included in the article/Supplementary Material, further inquiries can be directed to the corresponding author/s.

## Ethics Statement

The animal study was reviewed and approved by The Animal Experiments Committee of Kochi University of Technology.

## Author Contributions

DR and YKam conceived the study and designed the experiments. DR performed most of the *sox3* knock-in experiments. KO and KS performed the *sox11a* and *pax6a* knock-in experiments and qPCR analysis. YKaw conducted the whole-mount immunohistochemistry. YKun, TT, and YKam performed the remaining experiments. DR and YKam wrote the manuscript with input from the other authors. All of the authors approved the final version of the manuscript.

## Conflict of Interest

The authors declare that the research was conducted in the absence of any commercial or financial relationships that could be construed as a potential conflict of interest.

## Footnotes

1 https://ice.synthego.com
