# Supplementary material for: Efficient CRISPR-Cas9-Mediated Knock-In of Composite Tags in Zebrafish Using Long ssDNA as a Donor
Source: Front Cell Dev Biol. 2021 Feb 11;8:598634. doi: 10.3389/fcell.2020.598634 (PMC7928300; doi:10.3389/fcell.2020.598634)
Supplement: Supplementary file 1 [file Data_Sheet_1.pdf]

Supplementary Material

Efficient CRISPR-Cas9-Mediated Knock-In of Composite Tags in Zebrafish Using Long ssDNA as a Donor

Deshani C. Ranawakage†, Keita Okada, Kota Sugio, Yuya Kawaguchi, Yuki Kuninobu-Bonkohara, Takuya Takada, and Yusuke Kamachi*

School of Environmental Science and Engineering, Kochi University of Technology, Kami, Kochi 782-8502, Japan

*** Correspondence:**
Yusuke Kamachi
kamachi.yusuke@kochi-tech.ac.jp

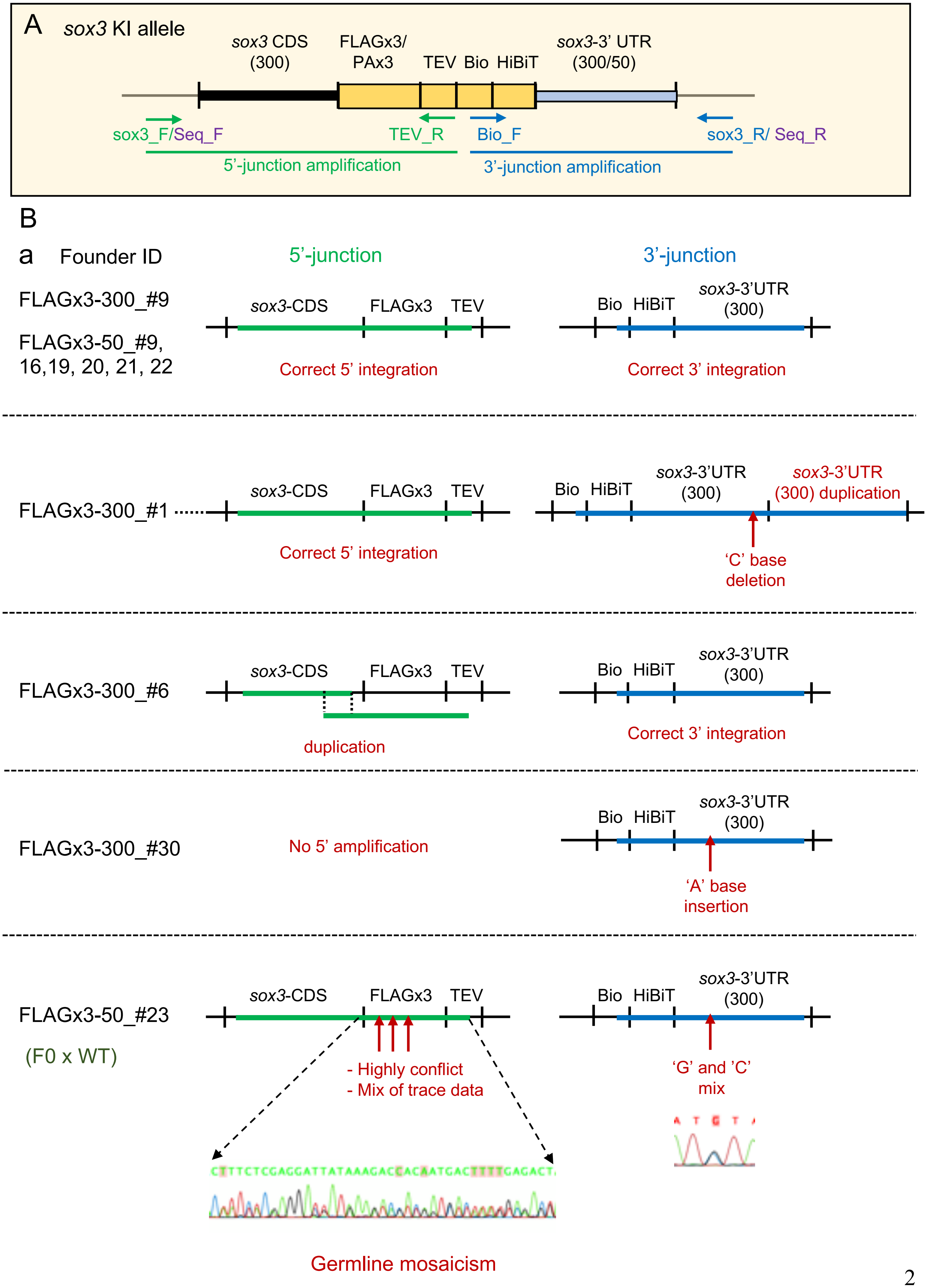
A
sox3 KI allele
sox3 CDS (300)
FLAGx3/ PAx3
TEV Bio HiBiT
sox3-3' UTR (300/50)
sox3_F/Seq_F
TEV_R
Bio_F
sox3_R/ Seq_R
5'-junction amplification
3'-junction amplification
B
a Founder ID
5'-junction
3'-junction
FLAGx3-300_#9
FLAGx3-50_#9, 16,19, 20, 21, 22
sox3-CDS FLAGx3 TEV
Bio HiBiT sox3-3'UTR (300)
Correct 5' integration
Correct 3' integration
FLAGx3-300_#1
sox3-3'UTR (300) duplication
'C' base deletion
FLAGx3-300_#6
duplication
FLAGx3-300_#30
No 5' amplification
'A' base insertion
FLAGx3-50_#23
(F0 x WT)
- Highly conflict
- Mix of trace data
'G' and 'C' mix
CTTTCTCGAGGATTATAAAGACCACAATGACTTTTGAGACT
Germline mosaicism

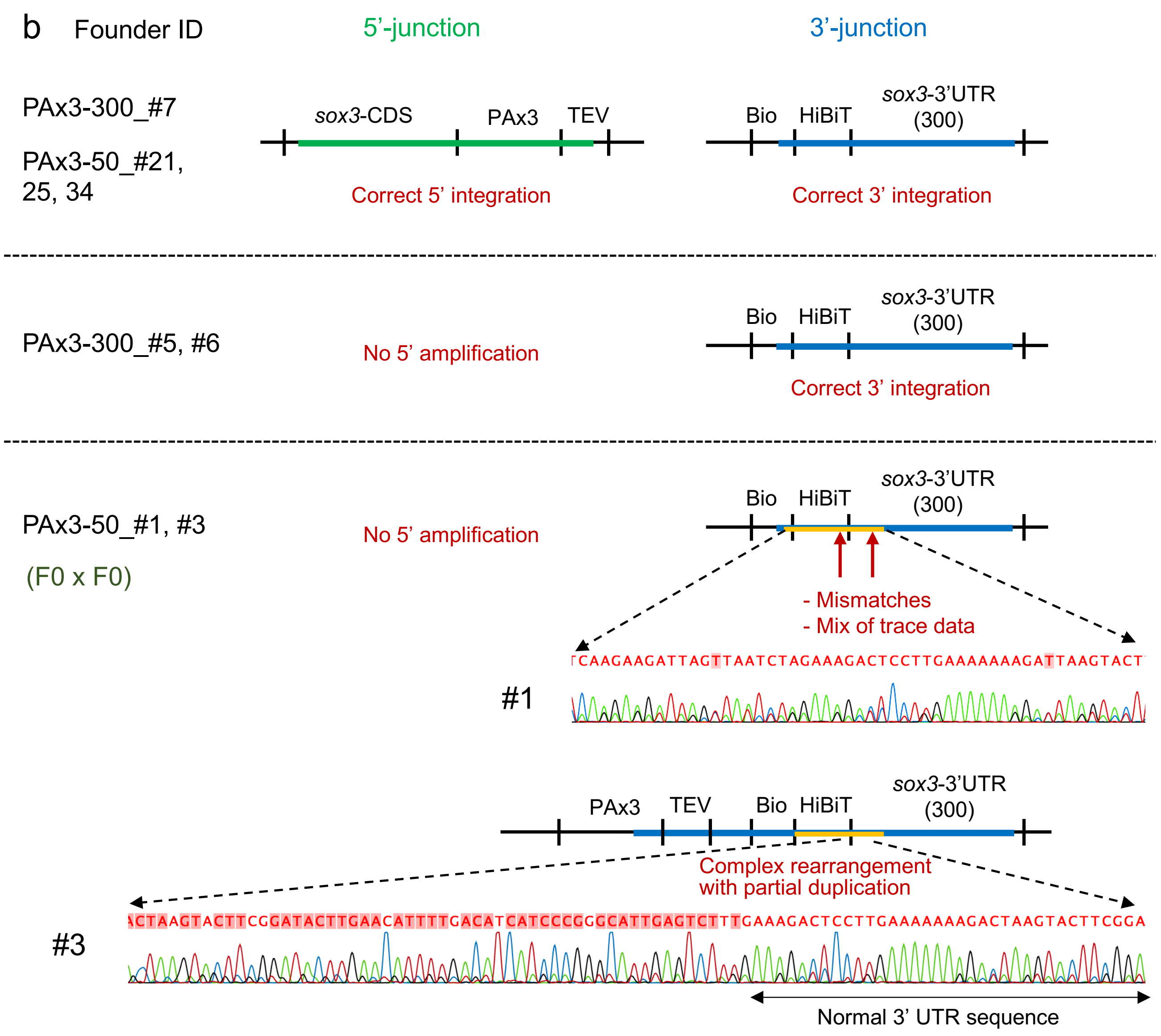

Supplementary Figure 1. Sequencing analysis of the knock-in events in F0 fish. (A) Schematic representation of PCR amplification of the 5' and 3' junctions of the *sox3* knock-in alleles. Positions of PCR and sequencing primers are illustrated in the upper box and the sequence of each primer is listed in Table S1. Genomic DNA was prepared from in-crossed or out-crossed F1 embryos and Sanger sequencing was performed using the junction PCR products. (B) The 5' and 3' junctions of the analyzed FLAGx3 (a) and PAx3 (b) composite tag alleles are indicated in green and blue, respectively, along with the Sanger sequencing chromatograms.

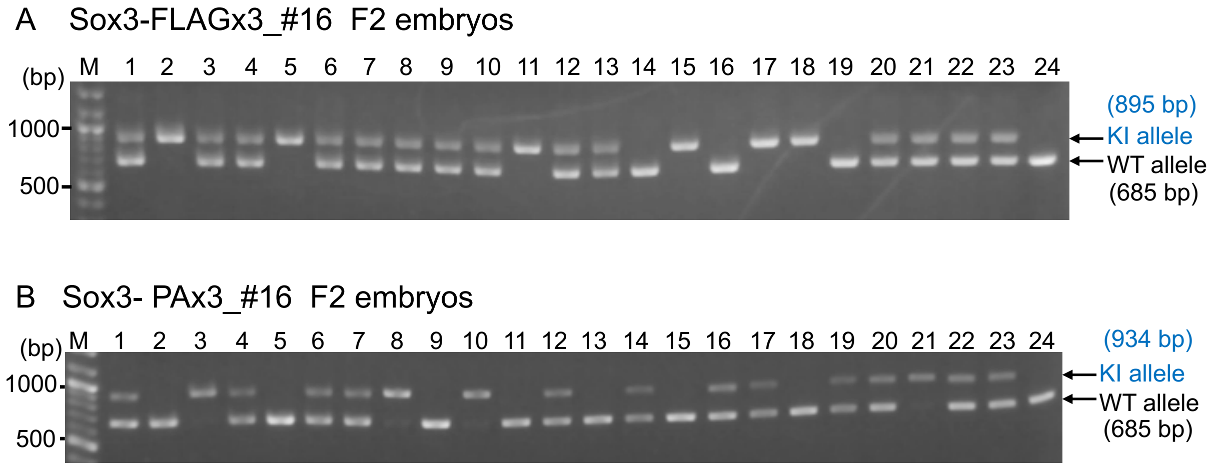

C Genotypic ratios of the F2 embryos

	KI / KI	KI / WT	WT / WT
Sox3-FLAGx3	25% (6/24)	58% (14/24)	17% (4/24)
Sox3-PAx3	17% (4/24)	50% (12/24)	33% (8/24)

Supplementary Figure 2. Transmission of the knock-in alleles from F1 to F2 generation. The heterozygous F1 knock-in fish derived from the FLAGx3-50_#16 and PAx3-50_#21 founders were crossed to obtain their F2 embryos. (A) The agarose gel electrophoresis image represents amplicons of PCR that was performed using genomic DNA from individual FLAGx3-50_#16 F2 embryos and the primers shown in Figure 6. (B) The agarose gel electrophoresis image represents amplicons of PCR that was performed using genomic DNA from individual PAx3-50_#21 F2 embryos. (C) Genotypic ratios of the F2 embryos.

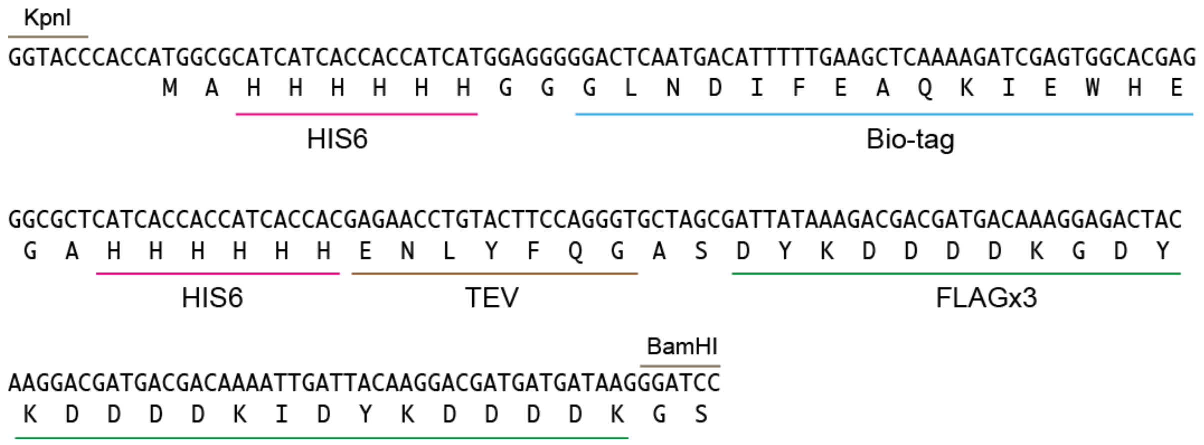

Supplementary Figure 3. Nucleotide and amino acid sequences of the HBH-FLAGx3 composite tag. The amino acid sequence of the HBH-FLAGx3 composite tag for N-terminal tagging is shown with its corresponding nucleotide sequences. The Kozak consensus and start codon sequences are preceded by the KpnI site and the FLAGx3 sequence is followed by the BamHI site to enable cloning into the pUC19 vector. TEV: TEV protease cleavage site; Bio tag: biotin ligase recognition site.

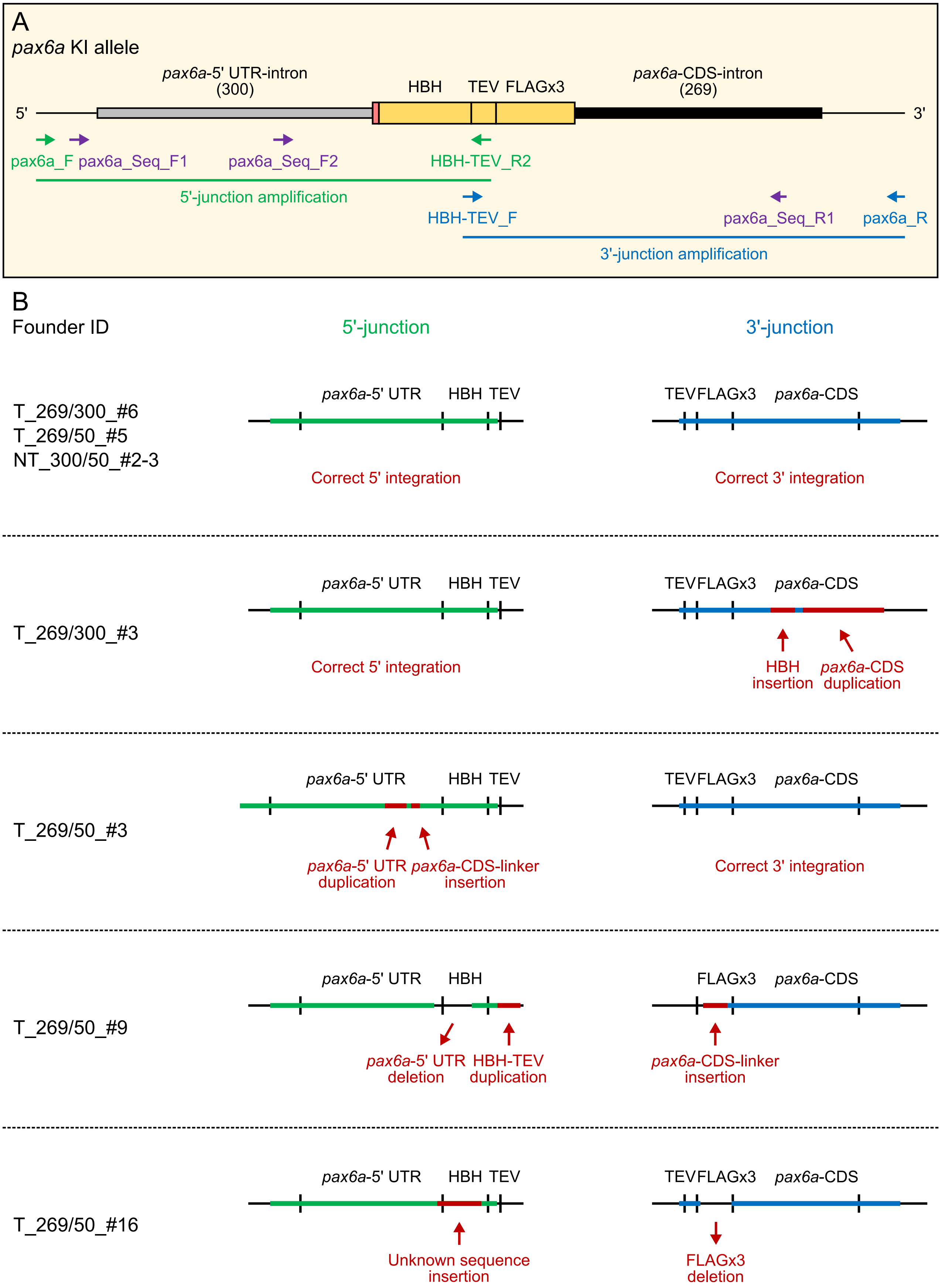
A
pax6a KI allele
pax6a-5' UTR-intron
(300)
HBH
TEV
FLAGx3
pax6a-CDS-intron
(269)
5'
3'
pax6a_F
pax6a_Seq_F1
pax6a_Seq_F2
HBH-TEV_R2
5'-junction amplification
HBH-TEV_F
pax6a_Seq_R1
pax6a_R
3'-junction amplification
B
Founder ID
5'-junction
3'-junction
T_269/300_#6
T_269/50_#5
NT_300/50_#2-3
pax6a-5' UTR
HBH TEV
TEVFLAGx3
pax6a-CDS
Correct 5' integration
Correct 3' integration
T_269/300_#3
HBH
insertion
pax6a-CDS
duplication
T_269/50_#3
pax6a-5' UTR
duplication
pax6a-CDS-linker
insertion
T_269/50_#9
FLAGx3
pax6a-5' UTR
deletion
HBH-TEV
duplication
pax6a-CDS-linker
insertion
T_269/50_#16
Unknown sequence
insertion
FLAGx3
deletion

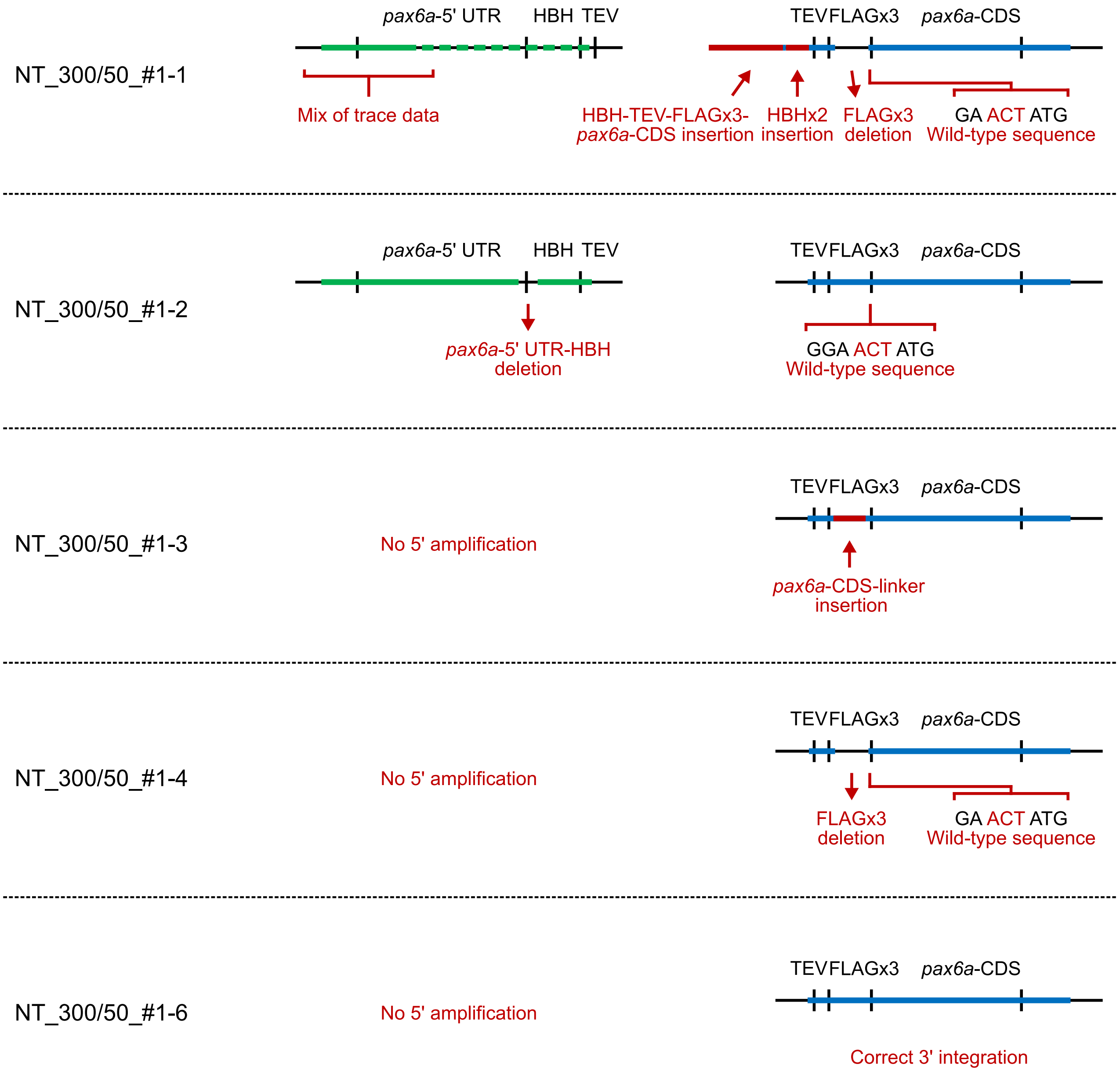

Supplementary Figure 4. Sequencing analysis of the knock-in events in F0 fish of the *pax6a* knock-in. (A) Schematic representation of PCR amplification of the 5' and 3' junctions of the *pax6a* knock-in alleles. Positions of PCR and sequencing primers are illustrated in the upper box and the sequence of each primer is listed in Table S1. Genomic DNA was prepared from in-crossed or out-crossed F1 embryos and Sanger sequencing was performed using the junction PCR products. (B) The 5' and 3' junctions of the analyzed composite tag alleles are indicated in green and blue, respectively.

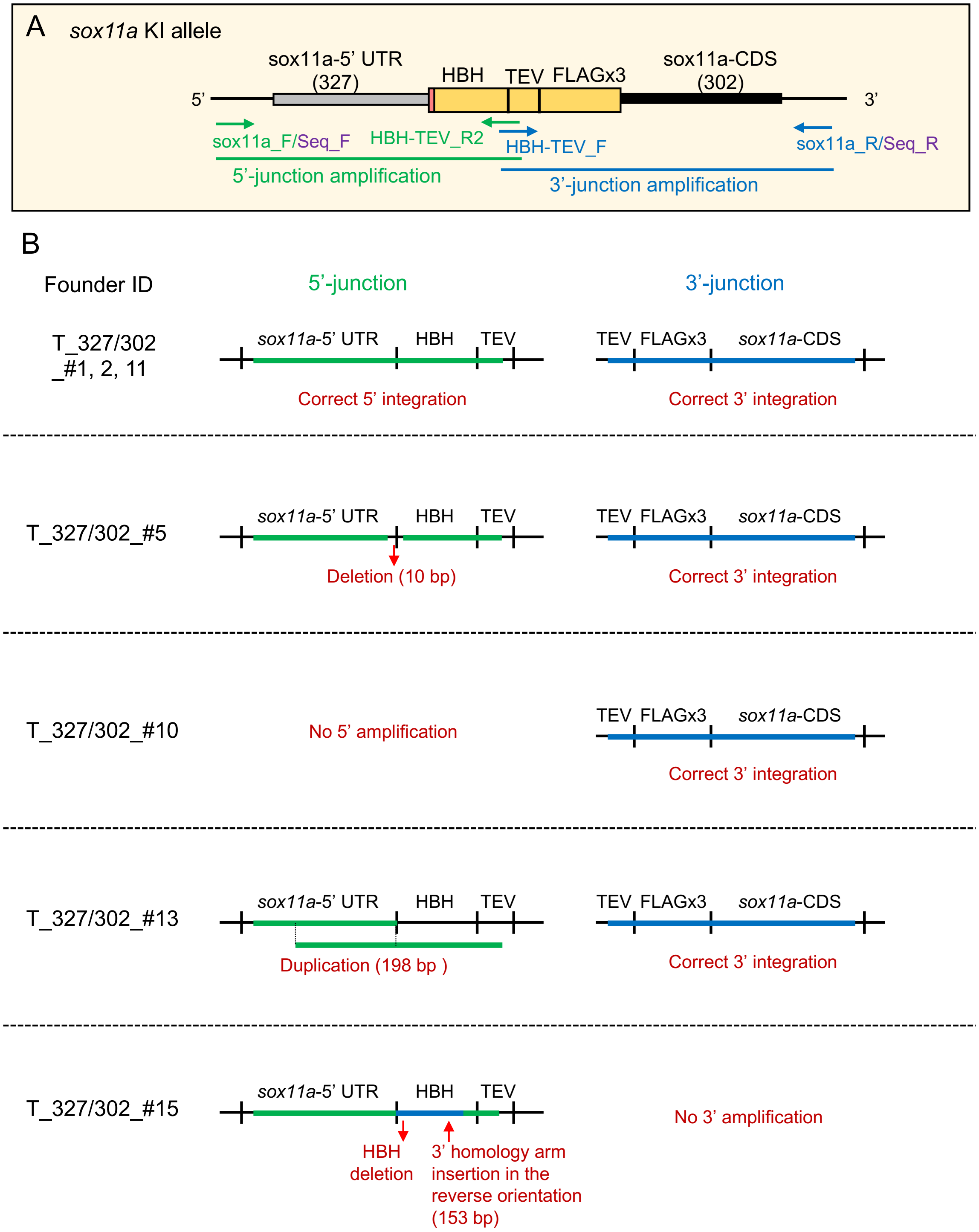

Supplementary Figure 5. Sequencing analysis of the knock-in events in F0 fish of the *sox11a* knock-in. (A) Schematic representation of PCR amplification of the 5' and 3' junctions of the *sox11a* knock-in alleles. Positions of PCR and sequencing primers are illustrated in the upper box and the sequence of each primer is listed in Table S1. Genomic DNA was prepared from in-crossed or out-crossed F1 embryos and Sanger sequencing was performed using the junction PCR products. (B) The 5' and 3' junctions of the analyzed composite tag alleles are indicated in green and blue, respectively.

Supplementary Table 1. PCR and sequencing primers.

PCR/sequencing	Primer name	Sequence
HMA PCR	sox3_HMA_F	ACTCCAGTCTACAGACCAGTC
	sox3_HMA_R	TTCAAGTATCCGAAGTACTTAGTC
Knock-in allele specific PCRs and sequencing	sox3_F/Seq_F	GCGGGACTTCAGTACCCAATGA
	TEV_R	TGGAAGTACAGGTTCTCACGCG
	Bio-tag_F	AAAGATCGAGTGGCACGAGG
	sox3_R/Seq_R	TGAACGTACTCTCCCTCCGT
	sox11a_F/Seq_F	TCTCTCCTTTAGTCTAACGGATCCTG
	sox11a_R/Seq_R	TGTATTTGTAGTCGGGGTAGTCAGC
	pax6a_F	AGCTAATGGGCCACTGAAGAG
	pax6a_R	CGGTCTTGGCCTACTGTGAC
	HBH-TEV_R2	ACCCTGGAAGTACAGGTTCTCG
	HBH-TEV_F	CACCATCACCACGAGAACCTGT
	pax6a_Seq_F1	GGCACAGACCAGGAACACATAC
	pax6a_Seq_F2	CCACCCGAGATCAGTTGGAAAC
	pax6a_Seq_R1	CACCACGAGGTTGTGCAG
ICE PCRs	sox3_F/Seq_F	GCGGGACTTCAGTACCCAATGA
	sox3_R/Seq_R	TGAACGTACTCTCCCTCCGT
	sox11a_F	AGTCTCTCGGCTTCCCTGATG
	sox11a_R	GAGCGCGTAAAGGGTTAAAGC
	pax6a_F2	CAGAGGTCAGGCTCAGCTAATGG
	pax6a_R2	AGGCAAAGAGGCTCCGTGAAAA
ICE Sanger sequencing	sox3_F/Seq_F	GCGGGACTTCAGTACCCAATGA
	sox11a_F(ICE)	CATTTTCCAGCGCTTCCCCAAG
	pax6a_R3	CACCACGAGGTTGTGCAG
PCRs for sox3 donor homology arms	sox3_5_CDS_EcoRI_F	CACGAATTCGCGTCCACGTACAGCAGCATGTC
	sox3_5_CDS_XhoI_R	GTCCTCGAGAATGTGTGTTAGGGGTAGCGTTCCGTTC
	sox3_3_UTR_XbaI_F	CACTCTAGAAAGACTCCTTGAAAAAAAGA
	sox3_3_UTR_PstI_R	GTCCTGCAGGAAATAGAGCCTCTTCACGAAGC
	sox3_3_UTR_50_PstI_R	GACCTGCAGTCAAAATGTTCAAGTATCCG
PCRs for sox11a donor homology arms	sox11a(-323)_PstI_F	GGGCTGCAGCAACATTCATAGCGCGCGGTTTG
	sox11a-5_UTR_KpnI_R	GGGGGTACCGGTGCCGTTGCCGTGCGTTG
	sox11a-GG-CDS_BamHI_F	GGGGGATCCGGCGGAATGGTGCAGCAAACGGACAAC
	sox11a(+302)_XhoI_R	GGGCTCGAGTGTTTCAACCGGAGTCTCTCGGC
	sox11a(-50)_PstI_F	GGGCTGCAGACGTACACACGGGTTGATAT
	sox11a(+50)_XhoI_R	GGGCTCGAGGCTTCTCTAGACATGCTGTC
PCRs for pax6a donor homology arms	pax6a_300bp_PstI_F	GGGCTGCAGAGTGCTATCACCAATCAGCAT
	pax6a_5_UTR-crAA_KpnI_R	GGGGGTACCGCCTTTGTATCCTCGCTGAAG
	pax6a_start-intron_BamHI_F	GGGGGATCCATGCCTCAAAAAGGTAAGTTAAGAC
	pax6a_EcoRI_R	GGGGAATTCAGGCAAAGAGGCTCCGTGAAAA
	pax6a_UTR_50bp_PstI_F	GGGCTGCAGAGAGTCTTCTCGTTATTGTAAACG
	pax6a_start-intron_50bp-EcoRI_R	GGGGAATTCGGCATATTGCTAAAAAGAGAAG

Supplementary Table 2. Primers and probes for qPCR.

qPCR target		Primer/probe name	Sequence	Annealing and extension temperature
sox3-PAx3-Bio-HiBiT	5' junction	sox3_F2	GTCCACGGCTCAGACCTACAT	62℃
		TEV_R2	CACCCTGGAAGTACAGGTTCTCA	
		sox3_5'_probe	CTCGAGAATGTGTGTTAGGGGTAGCGTTCC	
sox3-PAx3-Bio-HiBiT	3' junction	TEV_F	AACCTGTACTTCCAGGGTGGAG	62℃
		sox3_R(qPCR)	CGTACTCTCCCTCCGTTTCTCTTT	
		sox3_3'_probe	TCTAGATTAGCTAATCTTCTTGAACAGCCGCC	
sox11a-HBH-FLAGx3	5' junction	sox11a_F	TCTCTCCTTTAGTCTAACGGATCCTG	64℃
		HBH-TEV_R3	AAGTACAGGTTCTCGTGGTGATGG	
		sox11a_5'_probe	TACCCACCATGGCGCATCATCACCA	
sox11a-HBH-FLAGx3	3' junction	HBH-TEV_F2	CCATCACCACGAGAACCTGTACTT	64℃
		sox11a_R	TGTATTTGTAGTCGGGGTAGTCAGC	
		sox11a_3'_probe	TGATAAGGGATCCGGCGGAATGGTGC	
pax6a-HBH-FLAGx3	5' junction	pax6a_F	AGCTAATGGGCCACTGAAGAG	62℃
		HBH-TEV_R2	ACCCTGGAAGTACAGGTTCTCG	
		pax6a_5'_probe	AGGATACAAAGGCGGTACCCACCATGG	
pax6a-HBH-FLAGx3	3' junction	HBH-TEV_F	CACCATCACCACGAGAACCTGT	62℃
		pax6a_R	CGGTCTTGGCCTACTGTGAC	
		pax6a_3'_probe	AGGACGATGATGATAAGGGATCCATGCCTCA	
hesx1		hesx1_F	CCAAGCAGCCAACAGAGATCAA	60℃
		hesx1_R	ATGCTCGGCTTCACAAAAGCAC	
		hesx1_probe	TGCTTCCAGCAAATTGCCAAGCGGC	
